# Record linkage of population-based cohort data from minors with national register data: a scoping review and comparative legal analysis of four European countries

**DOI:** 10.12688/openreseurope.13689.1

**Published:** 2021-05-27

**Authors:** Julia Nadine Doetsch, Vasco Dias, Marit S. Indredavik, Jarkko Reittu, Randi Kallar Devold, Raquel Teixeira, Eero Kajantie, Henrique Barros

**Affiliations:** 1 EPIUnit, Instituto de Saúde Públicada, Universidade do Porto (ISPUP), Porto, 4050-600, Portugal; 2 Laboratory for Integrative and Translational Research in Population Health (ITR), Porto, 4050-600, Portugal; 3 INESC TEC -Institute for Systems and Computer Engineering, Technology and Science, Campus da Faculdade de Engenharia da Universidade do Porto, Porto, 4050-091, Portugal; 4 Department of Clinical and Molecular Medicine, Faculty of Medicine and Health Sciences, NTNU – Norwegian University of Science and Technology, Trondheim, NO-7491, Norway; 5 Finnish Institute for Health and Welfare, Legal Services, Helsinki, Finland; 6 University of Helsinki, Faculty of Law, Helsinki, Finland; 7 Faculty of Medicine and Health Sciences, NTNU – Norwegian University of Science and Technology, Trondheim, NO-7491, Norway; 8 Finnish Institute for Health and Welfare, Population Health Unit, Helsinki and Oulu, Finland; 9 PEDEGO Research Unit, MRC Oulu, University of Oulu and Oulu University Hospital, Oulu, Finland; 10 Children’s Hospital, Helsinki University Hospital and University of Helsinki, Helsinki, Finland; 11 Departamento de Ciências da Saúde Pública e Forenses e Educação Médica, Faculdade de Medicina, Universidade do Porto (FMUP), Porto, Portugal

**Keywords:** Record Linkage, Cohort data, Routine data, GDPR, Data processing, European Union, European Economic Area, Europe

## Abstract

**Background**: The General Data Protection Regulation (GDPR) was implemented to build an overarching framework for personal data protection across the European Union/Economic Area (EU/EEA). Linkage of data directly collected from cohort participants based on individual consent must respect data protection rules and privacy rights of data subjects. Our objective was to investigate possibilities of linking cohort data of minors with routinely collected education and health data comparing EU/EEA member states.

**Methods**: A legal comparative analysis and scoping review was conducted of openly online accessible published laws and regulations in EUR-Lex and national law databases on GDPR’s implementation in Portugal, Finland, Norway, and the Netherlands and its connected national regulations purposing record linkage for health research that have been implemented up until April 30, 2021.

**Results:** EU/EEA has limited legislative authority over member states. The GDPR offers flexibility for national legislation. Exceptions to process personal data, e.g., public interest and scientific research, must be laid down in EU/EEA or national law. Differences in national interpretation caused obstacles in cross-national research and record linkage: Portugal requires written consent and ethical approval; Finland allows linkage mostly without consent through the national Data Protection Supervisory Authority; Norway when based on regional ethics committee’s approval and adequate information technology safeguarding confidentiality; the Netherlands mainly bases linkage on the opt-out system and Data Protection Impact Assessment.

**Conclusions: **Though the GDPR is the most important legal framework, national legislation execution matters most when linking cohort data with routinely collected health and education data. As national interpretation varies, legal intervention balancing individual right to informational self-determination and public good is gravely needed for scientific research. More harmonization across EU/EEA could be helpful but should not be detrimental in those member states which already opened a leeway for registries and research for the public good without explicit consent.

## Introduction

Improving research on health services requires access to timely, complete, and accurate patient or organizational data ^1^. Data acquisition via patient registries in routine procedures and systems, or through population-based cohort studies represent important data collection tools for health research, health monitoring, disease prevention, diagnostics, and health improvement ^2– 6^. Routinely collected data are defined as systematic records of patient information gathered in registers/administrative databases such as (non-) electronic patient registries, hospital-based child health and social protection facilities, or educational institutions ^7– 9^. A cohort is a group of individuals sharing a statistical factor in a demographic study, and inviting the same individuals to repeated health examinations or other assessments is called a cohort follow-up assessment ^10^. Whereas routinely collected data cover comprehensive information on individual interaction with cross-divisional facilities, cohort data cover the distribution and determinants of health-related conditions and events in a specific population and explore the longitudinal relationship between a specific exposure and outcome providing high validity, accuracy, and effectiveness in development trends ^2, 3, 11– 14^.

Record linkage – the general merging of data from an individual or an event that are not available in a separate record into consolidate facts – is increasingly used to extend accessible data and to generate complete and comprehensive data for health service organization, policy making, and public health research at comparatively low expenses ^15– 18^. As it enables to respond to research questions that could not have been answered before the merge, it can be of paramount importance for research studies ^14, 17, 19^. Hence, linking routinely collected data with cohort data presents an asset to research in complementing comprehensive data of individuals on cross-sectoral service interaction with data on the associations between the characteristics in a specifically studied population ^5, 6, 14, 17, 20– 22^. Health and education data and their multidimensional outcomes are as social determinants of health a vital fragment for public health and biomedical research ^23^. Moreover, health and education data influence health service provision aiming to improve population health and responding to user expectations and their needs while reducing inequalities in health and responsiveness leading a basis for policy-making ^24^.

As health data are considered personal data, defined as “any information from which a person [a data subject] can be identified or potentially identified from”, the involvement of the General Data Protection Regulation (GDPR) is required. The GDPR along with the e-privacy directive, covering electronical communication ^25^ to be replaced by the e-privacy regulation ^26^, functions as the ultimate legal framework on data protection and data privacy that reinforces individual control of data subjects’ own data and their associated rights in a digitalized era ^25, 26^. The GDPR aimed to build an overarching framework to enhance transparency, support individual rights, and promote the growth of the digital economy ^27^. Its general principles include: Lawfulness, fairness and transparency; Purpose limitation; Data minimisation; Accuracy; Storage limitation; and Integrity and confidentiality ^28– 30^. After the GDPR was completed in May 2016 and came into effect on May 2018, its direct applicability as a regulation was enforced in all European Union (EU) member states, Iceland, Liechtenstein and Norway, which together comprise the European Economic Area (EEA).

Linking data records falls under data processing, which the GDPR defines as the acquirement and any subsequent operation in the handling of personal data to generate useful information ^1, 2^. The GDPR requires that any party that processes personal data to have at least one of the six legal bases: consent, performance of a contract, legitimate interest, vital interest, legal requirement, and public interest ^3^. Though not the only legal basis, when informed consent is sued as a legal basis in the sense of the GDPR, it should comply with the criteria of being informed, specific, freely given and demonstratable. Yet, the first two are difficult to meet in longitudinal cohort studies with volunteers where the research questions are broadly defined and several means, which can change over time, might be used to answer that broad range of questions. Moreover, in the context of health data, an additional legal basis is needed, which might be explicit consent but could also be another authorisation based on national law, as the GDPR left a margin in implementing the clauses on health data for the administration of the health care system, public health and research ^4^. As a result, the so called trialogue between the European Parliament, the Council and the European Commission ^31^ as substantial leeway was left to the member states ^32^. Hence, member states were in charge to implement or leave existing national legislation concerning the processing of health data for public health and research, including exemptions to the informed consent principle and direct applicable research exemptions ^32^.

Since the advent of the GDPR there has been considerable debate about the relation between the GDPR and research ^29^. Thus, this study investigates the possibilities of linking cohort data with routine health and education data comparing the European countries Portugal, Finland, Norway and the Netherlands for health research purposes.

## Methods

A legal analysis and scoping review based on PRISMA-ScR guidelines was conducted between September 15, 2020 until April 30, 2021.

### Data selection and eligibility criteria

***Countries.*** We selected four countries that are part of the EU/EEA which are located in the south, middle, and north of Europe to achieve geographic variability: Portugal, Finland, Norway and the Netherlands.

***Population group.*** We selected children as population group, by law called data subjects. Children were defined as a human being below the age of 18 years ^5^.

***Data type.*** Health (sensitive) and education (non-sensitive) data were included due to their distinct nature in data processing and importance for health research.

***Laws and regulations.*** All published laws and regulations on GDPR’s national implementation and connected national regulations in Portugal, Finland, Norway, and Netherlands purposing record linkage of cohort data from minors with routinely collected health and education data for health research that have been implemented up until April 30, 2021 were considered eligible.

### Exclusion criteria

***Laws and regulations.*** Register linkage studies that use only register data were not included in this analysis as it would be out of scope of the study’s objective. Although the GDPR regulation include, as personal data, all data derived from biological samples, such as those from biobanks, we excluded this data category as it deviates from the main objective of the study.

***Information sources.*** Openly online accessible databases EUR-Lex ^6^ and national law databases ( Table 1) were used. The databases were searched within the time period of September 15, 2020 – April, 30 2021.

**Table 1.** Main information sources.

	GDPR	Portugal	Finland	Norway	Netherlands
1	EUR-Lex [Online]. Available at: https:// eur-lex.europa.eu/eli/ reg/2016/679/oj	Diário da República [Online]. Available at: https://dre.pt/	GlobaLex [Online]. Available at: https://www.nyulawglobal.org/ globalex/Finland.html	Data ombudsman [Online]. Available at: https://tietosuoja. fi/en/impact- assessments	Verheid.nl [Online]. Available at: https:// wetten.overheid. nl/zoeken
2			DATABASES OF THE FINNISH PARLIAMENT [Online]. Available at: http://www.eduskunta.fi	Lovdata [Online]. Available at: https:// lovdata.no/	
3			FINNISH ELECTRONIC STATUTE SERIES. [Online]. Available at: http://www.finlex.fi	Datatilsynet [Online]. Available at: https:// www.datatilsynet. no/en/	
4			FINLEX - LEGAL DATA BANK [Online]. Available at: http://www. finlex.fi	Helsetilsynet [Online]. Available at: https:// www.helsetilsynet. no/en/)	
5			FINNISH LAW INFO [Online]. Available at: http://www. kauppakaari.fi and http://www. lakiverkko.com	Directorate of eHealth – Helsedata [Online]. Available at: https:// www.helsedata.no/en/	
6			EDILEX - LEGAL PORTAL [Online]. Available at: http://www.edilex.fi	Access to microdata [Online]. Available at: https://www.ssb.no/ en/data-til-forskning/ utlan-av-data-til- forskere	

### Search

EUR-Lex and national law databases were consulted to search for all significant laws on data protection and data privacy for the processing of health and education data. Cross-referencing between the articles allowed to link themes, terms and subjects. Instead of specific search expressions, key words were used when screening the law databases, searching for applicable laws and regulations and when verifying specific terms.

Examples of key words used in the Regulation (EU) 2016/679 [General Data Protection Regulation (GDPR)] – EUR-Lex: (“data processing” OR “processing” OR “data”) AND (“operation” OR “collection” OR “storage” OR “recording” OR “organization” OR “storage” OR “adaptation” OR “retrieval” OR “consultation” OR “use” OR “transmission” OR “dissemination” OR “alignment” OR “combination” OR “restriction” OR “erasure” OR “personal” OR “identification” OR “Information” OR “protection” OR “protect” OR “protection” OR “data subject” OR “consent” OR “minor” OR “children” OR “child” OR “subsidiarity” OR “parent” OR “legal person” “subject” OR “scientific research” OR “research” OR “health” OR “education” OR “security” OR “privacy” OR “routine” OR “register” OR “collect” OR “individual” OR “right” OR “principle” OR “duty” OR “duties” OR “population” OR “controller” OR “processor” OR “Pseudomization” OR “Anonymization” OR “data protection impact assessment” OR “Independent supervisory principle” OR “data minimization principle” OR “purpose limitation principle” OR “Storage Limitation Principle” OR “purpose” OR “statistical” OR “freedom” OR “burden” OR “Ethical approval” OR “Ethics” OR “Ethics Committee” OR “sensitive” OR “non-sensitive” OR “safeguarding” OR “provision” OR “administrative” OR “electronic record” OR “electronic” OR “personal information” OR “special categories”).

### Data analysis

We investigated the possibilities of linking routinely collected education and health data with cohort data comparing Portuguese, Finnish, Norwegian and Dutch law, and their interplay on record linkage purposing the conduction of research up until April 30, 2021. Data processing findings were analysed and compared across the selected countries from the EU/EEA enabling an overview of the main possibilities of record linkage ( Table 2).

**Table 2.** Evidence synthesis (based on the Joanna Briggs Institute (JBI) manual). GDPR=General Data Protection Regulation.

Scoping Review Details
**Scoping Review title:** Record linkage of population-based cohort data from minors with national register data: a scoping review and comparative legal analysis of four European countries
**Review objective/s**: Investigate possibilities of linking cohort data of minors with routinely collected education and health data comparing EU/EEA member states.
**Review question/s**: What are the possibilities of linking cohort data of minors with routinely collected education and health data comparing different EU/EEA member states?
Inclusion/Exclusion Criteria
**Population:** Children (minors), defined as a human being below the age of 18 years, were included as data subjects.
**Data type**: Health (sensitive) and education (non-sensitive) data were included due to their distinct nature in data processing and importance for health research.
**Laws and regulations:** All openly accessible published laws and regulations on GDPR’s national implementation and connected national regulations in Portugal, Finland, Norway, and Netherlands purposing record linkage of cohort data from minors with routinely collected health and education data for health research that have been implemented up until April 30, 2021 were considered eligible.
**Types of evidence source:** Openly online accessible databases EUR-Lex and national law databases (see Table 1) were used.
**Exclusion:** Register linkage studies that use only register data were not included in this analysis as it would be out of scope of the study’s objective. Although the GDPR regulation include, as personal data, all data derived from biological samples, such as those from biobanks, we excluded this data category as it deviates from the main objective of the study.
Evidence source Details and Characteristics
**Countries:** Portugal, Finland, Norway, and Netherlands
**Context:** Databases have been searched within the time period of September 15, 2020 – April, 30 2021.
**Details/Results extracted from source of evidence** (in relation to the concept of the scoping review)
**Synthesis of results:** see Table 3.

### Synthesis of results

All data (laws and regulations) that were included are listed in Table 3 and are marked throughout the results section with footnotes. Results were organized and clustered into six main themes: 1) Legal basis for research, 2) Legal basis for registries, 3) Representation of minors, 4) Opportunities to link, 5) Record Linkage with other data bases, and 6) Procedural conditions.

**Table 3.** Main involved laws. GDPR=General Data Protection Regulation.

European laws	GDPR–specific articles	Portugal	Finland	Norway	The Netherlands
General Data Protection Regulation (GDPR)- Regulation of the EU 2016/679	Article 4/2 GDPR; Article 4/5 GDPR; Article 4(11) GDPR; Article 4(13) GDPR; Article 4(14) GDPR; Article 4(15) GDPR	58/2019 Act, August 8, 2019; Article 31/4 of 58/2019 Act August 8 2019	Act on the Secondary Use of Health and Social Data (552/2019), March 13, 2019	Act of 15 June 2018 No. 38 on personal data (Personal Data Act) Lov om behandling av personopplysninger (personopplysningsloven)	General Data Protection Regulation Implementation Act, May 25, 2018 “Uitvoeringswet Algemene Verordening Gegevensbescherming” (UAVG)
Article 16 of the Treaty on the Functioning of the European Union, 2000	Article 6 GDPR; Article 6(1) GDPR; Article 6(1)(a) GDPR Article 6/1 b)-c) GDPR; Article 6(1)(e) GDPR; Article 6(1)(f) GDPR; Article 6(2) GDPR; 6(1)(e) GDPR	Article 80 of Portuguese Civil Code, 1966	Data Protection Act (1050/2018), December 5, 2018	Act of 20 June 2008 No. 44 on Medical and Health Research (Health Research Act) Lov om medisinsk og helsefaglig forskning (helseforskningsloven)	Aanpassingswet Algemene Verordening Gegevensbescherming, May 25, 2018
Recommendation CM/Rec (2019)2 of the Committee of Ministers to member states on the protection of health- related data	Article 9 GDPR; Article 9/1 GDPR; Article 9/1/a) GDPR; Article 9(2) GDPR; Article 9/2/i) GDPR; Article 9(2)ij) GDPR; Article 9(2)(h) GDPR; Article 9(2)(j) GDPR; Article 9/1/a) GDPR; Article 9/2/g GDPR; Article 9/2/i) GDPR; Article 9(4) GDPR	Constitution of the Portuguese Republic, 1976; Article 35 Constitution of Portugal, April 10, 1976	Act 556/1989	Act of 28 April 2017 No. 23 on Ethics and Integrity in Research (Research Ethics Act) Lov om organisering av forskningsetisk arbeid (forskningsetikkloven)	Afdeling 5 van Boek 7 BW
Recommendation No. R(97) 18 of Council of Europe	Article 86 GDPR	Organization and Functioning of the National Commission of data protection – 43/2004 Act, August 18, 2004	Laki sosiaali- ja terveystietojen toissijaisesta käytöstä, 552/2019	Act of 15 June 2018 No. 38 on personal data (Personal Data Act) Lov om behandling av personopplysninger (personopplysningsloven)	Article 7:457 lid 3 BW
Recommendation CM/Rec (2010)13 adopted by the Committee of Ministers of the Council of Europe on November 23, 2010	Recital 159 GDPR	Personal genetic information and health information – 12/2005 Act, January 26, 2005	Section 2, Act on the Openness of Government Activities (621/1999)	Act of 20 June 2014 No. 43 on Personal Health Data Filing Systems and the Processing of Personal Health Data (Personal Health Data Filing System Act) Lov om helseregistre og behandling av helseopplysninger (helseregisterloven)	Article 7:458 BW
Convention for the processing of individuals with regard to Automatic Processing of Personal data	Recital 26 GDPR	21/2014 Act, April 16, 2014- legal regime of clinical research	Laki viranomaisten toiminnan julkisuudesta, 621/1999	Act of 1 January 2021 No. 133 on Amendment in Personal Health Data Filing System Act / Lov om endringer i helseregisterloven m.m	Kamerstukken 31765
Article 2/b) of the Modernised Convention for the protection of individuals on processing of Personal data, of the 18th of May 2018	Article 89(1) GDPR; Article 89/2 GDPR	26/2016 Act, August 22, 2016	Tietosuojalaki, 1050/2018	Act of 21 June 2019 No. 32 relating to official statistics and Statistics Norway (Statistics Act) Lov om offisiell statistikk og Statistisk sentralbyrå (statistikkloven)	Article 41 Wet op het Centraal Bureau voor Statistiek
Regulation (EC) No 1338/2008, December 16, 2008	Article 5(1) (b) GDPR ; Article 5/e) GDPR; Article 5 (2) GDPR	Regulation no. 1/2018 by the National commission of data protection, October 16, 2018	Section 1, Data Protection Act (1050/2018), January 1, 2019	Regulation on medical quality health registers - Forskrift om medisinske kvalitetsregistre, of June 21 2019, entered into force on September 01, 2019	Article 7:465 BW
Paragraph 1 of Recommendation No. R (97) 18, September 30, 1997	Article 8 GDPR; Article 8 (1)	Article 31/4 of Law nº 58/2019 Act, August 8, 2019, The Portuguese Data Protection Act	Laki lääketieteellisestä tutkimuksesta, 488/1999	Act of 20 June 2008 No. 44 on Medical and Health Research (Health Research Act) Lov om medisinsk og helsefaglig forskning (helseforskningsloven)	Article 5, GDPR Dutch implementing Act
Article 3/c) of Regulation (EC) no. 1338/2008, December 16, 2008	Article 35 GDPR; Article 35/1 and 2 GDPR; Article 35/3/b GDPR	Law nº 21/2014, of 16 April	Section 2(1) of Medical Research Act (488/1999) October 1, 2010	Forskrift om barn mellom 12 og 16 år sin rett til selv å samtykke til deltakelse i medisinsk og helsefaglig forskning	Article 46 Dutch implementing Act
Paragraph 3.3 of the Recommendation No. R (97)18, September 30, 1997	Article 36 GDPR; Article 36(9) GDPR	Law nº. 12/2005 of 26 January on Personal genetic information and health information.	Section 6, Medical Research Act (488/1999), October 1, 2010	Act of 20 June 2014 No. 43 on Personal Health Data Filing Systems and the Processing of Personal Health Data (Personal Health Data Filing System Act) Lov om helseregistre og behandling av helseopplysninger (helseregisterloven)	
Paragraph 6 of Recommendation CM/Rec (2019)2, March 27, 2019	Recital 32 GDPR	Decree-Law No. 97/95, May 10	Section 44, Act on the Secondary Use of Data (552/2019)	Statistics act §14, Act of 21 June 2019 No. 32 relating to official statistic and Statistics Norway (Statistics Act).	
Article 4/3 of 12/2005 Act, January 15, 2005	Recital 40 GDPR	Law nº 81/2009, of 21 of August		Act of 21 June 2019 No. 32 relating to official statistics and Statistics Norway (Statistics Act) of 21 June 2019 Lov om offisiell statistikk og Statistisk sentralbyrå (statistikkloven)	
Paragraph 6 of Recommendation CM/Rec (2019)2, March 27, 2019	Recital 162 GDPR	Law nº 53/2017 of 14 July		Regulations to the Statistics Act/ Forskrift til statistikkloven av Dec 11th 2020 No 2731 (FOR-2020-12-11-2731) Forskrift til statistikkloven (statistikkforskriften)	
Article 3/c) of Regulation (EC) no. 1338/2008	Recitals 33 GDPR	Law 22/2008, of 13 May		Act of 1 January 2021 No. 133 on Amendment in Personal Health Data Filing System Act / Lov om endringer i helseregisterloven m.m.	
Regulation (EU) 2016/679 of the European Parliament and of the Council, April 27, 2016	Recital 50 GDPR	Article 124 of Portuguese Civil Code		Act of 20 June 2008 No. 44 on Medical and Health Research (Health Research Act) Lov om medisinsk og helsefaglig forskning (helseforskningsloven)	
Regulation (EU)2018/1725; Article 29	Recital 54 GDPR	Article 8º / 3, Decree Law nº 131/2014 of 29 of August		Act of 20 June 2014 No. 43 on Personal Health Data Filing Systems and the Processing of Personal Health Data (Personal Health Data Filing System Act) Lov om helseregistre og behandling av helseopplysninger (helseregisterloven)	
43/2004 Act	Recital 32 GDPR	Article 6/3 ''Código dos regimes contributivos do sistema previdencial de segurança social''		Health Research Act §17, §9 and 10	
Article 4/3 of 12/2005	Recital 157 GDPR	14/2013 Decree- law, January 28, 2013;		Forskrift om barn mellom 12 og 16 år sin rett til selv å samtykke til deltakelse i medisinsk og helsefaglig forskning	
Working Party (A29WP)	Recital 159 GDPR	Article 99/1 of the 4/2007 Act, January 16, 2007			
	Recital 4 GDPR	Article 3/1 of the 'Despacho n.º 1774-A/2017, February 24, 2017			
		Article 6/5, of 22/2008 Act, of 13 May			
		Article 2º of 22/2008 Act, of 13 May			
		Article 62/2 of 58/2019 Act August 8, 2019			
		Article 9, Law 53/2017, of 14 July			
		Article 16, Law 53/2017, of 14 July			
		Article 13, Law 53/2017, of 14 July which Creates and regulates the National Cancer Registry (National Oncologic Registry Act)			
		Article 1/1 of 21/2014 Act, April 16, 2014			
		Article 6 of the 21/2014 Act, April 16, 2014; Article 6/1/b) and Article 6/1/d) of the 21/2014 Act, April 16, 2014; Article 6/1/e) of 21/2014 Act, April 16, 2014			
		Article 16/1 of the 21/2014 Act, April 16, 2014			
		Article 35 GDPR and Regulation 1/2018 CNPD			
		Article 4/4 of 12/2005 Act, January 26, 2005			
		Article 124 of Portuguese Civil Code			
		Official Gazette No. 274/1966			
		Decree-Law No. 47344			
		Decree-Law No. 97/95, May 10			

## Results

### GDPR

The GDPR operates as the chief legal framework for the protection of personal data and data privacy among countries who are part of the EU/EEA given its direct applicability as a regulation, while granting member states a significant margin of discretion in its implementation. The Helsinki declaration and other related declarations also play a role in the complete application of the GDPR ^33^. Data protection, data privacy, and legal contexts for research purposes are constructed on each legal setting of EU member states and countries of the EEA. However, given the precedence of EU law principle, as the GDPR is hierarchical higher, its appliance stands above member state law. Partner countries of the EEA agreement are bound by the GDPR in the same manner as EU member states. As a legally binding document it provided technical guidance to all entities that are bound to enforce it ^28, 30, 34^.

***Legal basis for research.*** The GDPR allows three types of research exception conditional on the obligations inflicted by Article 89(1): i) Exceptions to principles and lawful grounds for data processing; ii) exceptions to data subject rights; iii) national law implementation by member states ^35^. Member states may disclose official documents in accordance with member state law and grant access to official registry data under their member states law ^7^.

The GDPR grants the processing of sensitive data with a scientific research purpose under conditions like professional secrecy, Articles 6º and 9º of the GDPR shall be read and interpreted together in this regard. The GDPR states that scientific research and statistical purposes are connected, as statistical results may be used to achieve scientific outcome ^8^. While the first provides the six general legal bases ^9^ the second sets out a list of ten additional specific conditions, permitting the lawful processing of sensitive data ^10^. Scientific research is considered a legitimate reason and allows the compressing of the rights of a data subject ^11^. The purpose limitation principle needs to be applied, which enforces that personal data can be collected for a specified, explicit, and legitimate purpose ^12^. However, the GDPR provides for possible deviations from this principle: further processing for scientific research purposes, when respecting certain safeguards ^13^, benefits from a presumption of compatibility with the initial purposes ^14^. Article 5º provides six principles on personal data processing which inter alia include the purpose limitation principle that data should be “collected for specified, explicit and legitimate purposes” and data minimization principle that is to “limit [to the necessary purpose] […] for which they are processed”.

The GDPR provides safeguards and derogations from data subjects rights when data is processed for scientific research and statistical purposes including sensitive data ^15^. The use of information to characterize a collective phenomenon in a given population and the processing of personal data for statistical, scientific, or historical purposes is permitted subject to appropriate safeguards and the adoption of technical and organizational measures (e.g., pseudonymization, anonymization) ^16^. The processing of community statistics on public health and on health and safety at work is granted ^17^. It is prohibited to take decisions or actions related to a specific individual ^18^. Public health interest is defined as all essentials that are linked to health (e.g., health status) ^19^. If a statistical analysis cannot be carried out with anonymized data, collected data for a certain purpose must be anonymized as soon as possible ^20^. Pseudonymization may also be an adequate measure where the purposes of the research can be fulfilled in that manner.

***Legal basis for registries.*** The GDPR specifies that accessing data falls under the overall term of data processing ^21^. The GDPR established an inclusive explanation of the personal data processing for scientific research acknowledging the importance of data collection for research purposes in registries ^22^. A registry is a data collection system where official records are kept. In order to access data, either an explicit informed consent provided in an oral or written format is required, or the anonymization of personal data of the data subject ^23^. The acquisition of personal data must also be based on a specific form of consent.

***Representation of minors.*** Data subjects that are considered minors, have no legal capacity and are in need of a higher protection by law ^24^. Therefore, the legal guardian or representative authorizes the processing of personal data or the anonymization of data ^25^ on behalf of the data subject ^26^. Under GDPR the minimum of 13 years applies but only for Information on Society Services defined as “any service normally provided for remuneration, at a distance, by electronic means and at the individual request of a recipient of services ^36^. Apart from that, the age definition of a minor varies across the country-specific contexts.

***Opportunities to link.*** The GDPR generally prohibits the processing of sensitive data unless certain conditions are met [see legal basis for research]. The collection of routine data is part of the classification of substantial public interest and is permitted but not freely accessible and cannot be shared by third parties ^27^. Routinely collected data can contain non-sensitive (e.g., education data) and sensitive data (e.g., clinical information). If the latter applies, the rules of sensitive data collection are followed.

Health data is considered sensitive data and requires an explicit consent from the data subject whenever consent is the legal basis for processing. Health information collected for health research namely based on consent should also comply with other general data protection principles including the storage limitation principle ^28^. The storage limitation principle follows the idea of keeping the data for not longer than necessary “for the purposes for which the personal data are processed” ^29^. It defines that if the time of storage is unknown, an adequate condition for data storage has to be granted ^30^. The GDPR further states that “personal data may be stored for longer periods insofar as the personal data will be processed solely for archiving purposes in the public interest, scientific or historical research purposes or statistical purposes in accordance with Article 89(1)” ^31^.

Education data is non-sensitive information collected on education (e.g., educational level, grades) by schools. The collections of non-sensitive information follows for example the same category of protection as the collection of personal information ^37^. Access to non-sensitive data is less limited as it implies lower risks in relation to the rights and freedoms of the data subject.

***Record linkage with other databases.*** Linking cohort data with routine health and education data requires adherence to data privacy protection practices and guidelines. Data privacy protection practices include the provision of an informed explicit consent. Three lawful grounds on sensitive data processing are of main importance for the objective of linking routine health and education data with cohort data: i) explicit consent; ii) reasons of public interest in public health; iii) need for scientific, historical, and statistical purposes ^32^.

***Procedural conditions.*** The GDPR established the independent supervisory principle which defines that the data controller and the data processor must guarantee that the data processing meets the terms of the data protection rules ^33^. In defined circumstances, regarding processing operations likely to result in a high risk, the data controller has to follow a Data Protection Impact Assessment (DPIA), which implies to carry out an assessment of the resulting risks for data subjects as well as of the appropriate measures to mitigate them, and requires to seek advice from the data protection officer ^34^ (DPO). The data controller defines the purposes and the essential means of the processing of personal data ^37^ while the data processor acts on behalf of the data controller, following its documented instructions.

### Portugal

***Legal basis for research.*** In Portugal, the national implementation of the GDPR was finalized on August 8, 2019 ^35^. Typically, as per Article 6º/1 GDPR, the processing of personal data for research purposes is grounded either on the consent of data subjects (a) the performance of a task in the public interest (e) or the legitimate interests of the data controller f). The use and reuse of data for scientific research is not the subject of a dedicated legal instrument regulating it in a comprehensive way. However, the Clinical Research Act ^36^, the Health Information Act ^37^, the data protection act, and several other instruments contain provisions regulating research related matters. The Health information Act clarifies that health information belongs to data subjects, the health system being its custodian, and can only be used for health care or health related research, except where otherwise provided by law. Access to health records is granted to the data subject, or to a third party with the data subjects’ explicit written consent, through the intermediation of a medical doctor.

For the processing of health personal data held by the national health system in research, explicit written informed consent is required. Without consent, access to health information is allowed for research purposes only if anonymized. The same written informed consent requirement applies do biobanks samples and data, with the exception of retrospective research studies or the collection of epidemiological data, as consent cannot (reasonably) be obtained due to data quantity, number or age of human subjects or similar reason. Therefore, consent requirement may be disregarded only in exceptional circumstances, namely in the case of retrospective use of samples or in special situations where it is impossible to obtain consent. And only through legal interpretation this exception provided for biological materials and deoxyribonucleic acid (DNA) samples may be extended to routinely collected data in general ^38^. The Health Information Act further specifies provisions on the creation and operation of biobanks ^39^ as well on the processing of genetic information for the constitution of genetic databases, which will not be analysed in this study.

The Clinical Research Act adopts a broad definition of clinical research, comprising a non- exhaustive list of clinical trials and clinical studies ^40^, including certain observational studies, and requiring the informed consent from data subjects. The obligation to collect an informed consent for the participation in non-interventional clinical studies can exceptionally be derogated by determination of the Competent Ethics Commission ^41^; however, the consent for the processing of personal data may only be disregarded under the exceptional circumstances stated above ^42^. Similar conditions may be found in the legislative acts creating the existing disease registries. Therefore, in Portugal, health-related scientific research essentially relies on consent, as the legal grounds for the processing of personal data. Following the GDPR approval, the Portuguese new data protection act ^43^ timidly touched upon the subject of scientific research, exception made to the possibility of giving consent to certain areas of research (as in recital 33, GDPR). 

***Legal bases for registries.*** In what concerns registries, the recent legislation implementing the GDPR provides a specific provision allowing the processing implied in the organization of centralized health data bases or registries, based on a unique platform, for legitimate purposes under GDPR or national law, provided that the information security requirements resulting from the GDPR are ensured. There is no legal instrument dedicated to regulating the creation of registries, in general, nor registries in the specific the field of health. Notwithstanding the above, several health-related systems and (disease) registries were created under a specific legal act respectively, such as National Epidemiologic Surveillance Information System ^44^ and National Oncologic Registry ^45^.

However, a National Statistical System was established by law ^46^, mirroring the European Regulations on statistical agencies, having generated a comprehensive set of registries in various fields, mostly centralized at the National Statistics Institute. The statistical authorities (including the National Statistics Institute) may require the compulsory provision, from any services or bodies, individuals and legal entities alike, of data relevant for the production of official statistics. The National Statistical System is coordinated by the Superior Council of Statistics, which integrates representatives from the statistical authorities, among other entities, including a representative from the data protection supervisory authority.

The Directorate-General for Education and Science Statistics provides databases in the area of education and science and technology. For research purposes, it also provides the request for accreditation of researchers for access to National Statistics Institute resident databases, in accordance with a Protocol established with the National Statistics Institute and the Foundation for Science and Technology.

***Representation of minors.*** Under Portuguese law, a natural person below the age of 18 years is considered a minor and is legally vulnerable and benefit from greater protection. Their limited legal capacity must be suppressed through guardianship by holders of parental responsibility ^47^. The legal guardians or holders of parental responsibilities need to provide the consent and can authorize the personal data processing on behalf of the data subject. The age for consent has not been set in the Implementation Act exception made to implement Article 8º GDPR where it was established at 13 years. Additional requirements may apply in specific contexts where the opinion of minors and incapacitated adults must be considered as a determining factor, in accordance with their age, degree of maturity and capacity for understanding, their opposition must be respected, and at least their assent shall be previously obtained ^48^.

***Opportunities to link.*** Portugal has significant resources at its disposal for the collection and linkage of data such as the Ministry of Health, including an e-Health national agency ^49^, the Ministry of Education, the Directorate-General of Health and the National Institute of Statistics. Such entities may undertake decisions on data access and sharing to extent allowed by the applicable legal framework, considering the protection of personal data and the safeguarding of the public interest ^30^.

Several unique identifiers, which allow the identification of an individual, are specified by law ^50^ for numerous purposes, for example: social security number ^51^, tax number ^52^, user number for the National health Service ^53^, as well as the civil identification number. Those identifiers are contained in the electronic citizens Identification (ID) card through which citizens may exercise data subjects’ rights, in particular their access right, in several contexts. For instance, patients can access their electronic health records data through the electronic health registry in the citizen ´s portal of the National Health Service using their citizen card for authentication purposes. In the health sector an extensive network of Information Technology (IT) systems and databases exist under the supervision of the Ministry of Health.

Several legal provisions define the interconnection and interoperability (at the national and European level) between databases hosted in public entities for specific cases and purposes, including for research ^35– 37^. Portugal participates in European eHealth Digital Service Infrastructure, allowing the sharing of summary records and prescriptions, and has implemented a national system for the electronic reporting of laboratory notifications for infectious diseases, which ensures the interoperability between the laboratories IT system and National Epidemiologic Surveillance Information System. In specific contexts the use of sensitive data bases was permitted by law for research purposes like was the recent case of anonymized data from patients diagnosed with coronavirus disease 2019 (COVID-19) collected through the Surveillance Information System during the pandemic.

***Record linkage with other databases.*** Linking routinely collected health and education data with cohort data is feasible for research purposes, particularly based on consent, provided that the data subjects’ rights, the general principles and certain requirements of data protection law are respected **.** If data processing involves linkage between special categories of data, such as health data, and non-sensitive data, the legal regime of sensitive data must be complied with, without exclusion of special additional requirements rendered applicable by law, for instance to the usage of specific registries data.

The Law on the National Statistical System provides that individual statistical data relating to natural persons may not be supplied unless the data subject has given his or her explicit consent or with the authorisation of the Statistical Council ^54^. Otherwise, individual data may still be shared with universities and other recognized research organizations for scientific purposes, if data is anonymized ^55^ and a contract is in place between the statistical authority and the requesting research entity, establishing the necessary technical and organizational measures required to ensure the confidentiality of data and the respect for the purpose limitation principle. In order to pursue its mission of public interest, the National Institute of Statistics is allowed by law to carry out the processing of personal data, including sensitive data, and data linkage, namely with other statistical authorities.

In the case of the National Oncologic Registry, a centralized national registry of all cancer patients diagnosed and/or treated in Portugal, allowing for the epidemiological surveillance and research as well as the monitoring the effectiveness of medicines and medical devices, it may interconnect with other databases. Also, the interconnection between non-exclusive health databases is allowed ^56^, through the Public Administration Interoperability Platform ^57^ as well as the interconnection with other European oncology registries, in accordance with the standards and guidelines defined at the European level for this purpose ^58^. Access for research purposes from third parties to the data contained in the National Oncologic Registry electronic platform may be authorized by a special committee chaired by the director of the National Program for Oncological Diseases, “provided that, cumulatively, they are duly anonymized, it is not possible to identify the respective holder, and the public interest of the study is recognized” ^59^.

It should be noted that while the GDPR allows member state law to impose or maintain special conditions and limitations in what concerns the processing of health data ^60^, as well as prior consultation and authorization from the supervisory authority in relation to processing for the performance of tasks in the public interest, “including social protection and public health” ^61^, none of the relevant national provisions existing prior to the GDPR were subject to revision since the Regulation was put into effect. 

***Procedural conditions.*** Portuguese law defines clinical research as a systematic study that analyses the distribution or consequence of features of health which includes personal data and requires the respect of human dignity ^62^. Specific requirements for the conduction of a general clinical study must be met ^63^: 1) comprehensive study information and prior informed consent ^64^; 2) guaranteeing liability protection ^65^; 3) compliance with ethic committee authorizations ^66^; 4) special committees’ authorization; 5) the performance of a data protection impact assessment may also be required ^67^, in which case the controller shall seek the advice of the data protection officer. A DPIA may be subject to prior consultation of the supervisory authority ^68^.

### Finland

***Legal basis for research.*** In Finland there are several laws concerning the scientific research and access to public data in addition to the GDPR. The Data Protection Act ^69^ specifies and supplements the GDPR as national implementation of the GDPR ^70^. The Data Protection Act includes sections that specifies the general conditions to be fulfilled for the scientific research. Medical Research Act ^71^ is applied to medical research and clinical trials ^72^ alongside with other legislation. The Medical Research Act defines specific procedures for medical research, like necessity of informed consent of research subjects ^73^. The Act on the Openness of Government Activities ^74^ contains provisions on the right of access to official documents in the public domain, officials’ duty of non-disclosure, document secrecy and any other restrictions of access that are necessary for the protection of public and private interests ^75^.

The Act on the Secondary Use of Health and Social Data ^76^ was finalized on April 26, 2019. The main purpose of this Act is to simplify processing and access to personal social and health data for steering, supervision, research, statistics and development in the health and social sector. A secondary objective is to assure legitimate expectations of the data subject and its rights and freedoms for personal data processing ^77^.

In practice, the legal ground for scientific research in Finland can be based on all sensible options specified in the GDPR Article 6: consent (a), legal obligation (c), scientific research in the public interest (e) or legitimate interest (f). Typically processing of the personal data is based on scientific research in the public interest (e) and in fewer cases on consent (a). Article 9 exemptions applied for processing of special categories of personal data are typically necessity for scientific research (j) or explicit consent (a) and sometimes necessity for reasons of the public interest in the field of public health (i) or necessity for reasons of substantial public interest (g).

Ethical principles defined in the Oviedo Convention and Declaration of Helsinki are implemented in the Medical Research Act. Informed consent is required for participation in medical research, but it is distinguished from explicit consent as only option for legal basis. Therefore, scientific research in the public interest may be valid legal basis even the informed consent is required for the ethical reasons. In this case informed consent may act as additional safeguard.

***Legal basis for registries.*** Finnish national registries are based on national legislation that stipulates conditions for processing personal data in these registries. Legal basis is legal obligation (Article 6 (c)) or task carried out in the public interest (Article 6 (e)). The processing of personal data in these registries for scientific research is allowed by national legislation. The Finnish institute for health and welfare preserves or manages centralized registries ^78^ that contains complete database on all Finnish and foreign people that have used public health and social care services in Finland.

***Representation of minors.*** Under Finnish legislation everybody who is under 18 is considered a minor. However, if the minor is 15 years or older, their own consent is sufficient for participation in the research, if consent is needed. Even if participation requires the approval of parent or legal representative, minors primarily give their own consent ^79^.

***Opportunities to link.*** In Finland every citizen and permanent resident has a unique national identification number for all registrations. It is provided at birth or at immigration and reported without necessary consent to the registries as defined by law ^38, 39^. The main purpose of registries is administration, monitoring, and quality assurance ^39^. Registry data can be used for further purposes such as scientific or historical research or for statistical purposes ^80^.

Accordingly, with the Data Protection Act Section 29, the personal identity code may be processed if the data subject has given consent to it or for the scientific or historical purposes or statistical purposes ^81^. It is permitted to retrieve data from each of the registry-keeping authorities (e.g., health, social information) for research purposes under special circumstances. If possible, pseudonymized or non-individual-level data for medical research is preferred by authorities. Remote access to pseudonymized data is commonly granted. Consent is not required for individual level data.

Health and Social Data Permit Authority FinData issue permits for social and health data for the scientific research when data in needed from registers of multiple public data controllers, single private data controller or if public body, like the Finnish National Institute of Health and Welfare, have transferred permit authority to FinData ^40^ ^82^. If data is needed from other registries, then usually permits are issued by relevant authorities. Data Protection Ombudsman controls the processing of personal data and delivers permit related statement for the Data Permit Authority if requested.

***Record linkage with other databases.*** Consent is not mandatory for record linkage, but if consent is required for ethical reasons, then consent must include the record linkage. Typically, routinely collected health and education data with cohort data can be linked if a consent is provided. Access to identifiable data can be granted in limited cases if necessary, for research and if data security is sufficiently high. That implies that either the researcher have already the identification numbers in their own cohort, or researcher will link additional data to their dataset (e.g. medical records from the hospitals) ^40, 41^. If FinData grants authorization, then FinData is also responsible for the record linkage in the most cases.

FinData authority takes ultimate responsibility for all research use of the data in their registry if data is required by a single registry holder ^38, 42^. The authorization and processing of registry data for research purposes requires charges ^40^. Information from each registry can be shared and linked to the information from other registries in other Nordic countries. Data from health registries can be shared with research collaborators in other EU/EEA countries ^40^.

***Procedural conditions.*** Ethical review is required e.g., if a study involves an intervention in the physical integrity in clinical research, a study deviates from the principle of informed consent or review is needed for scientific publication. If a research study uses only register-based information, the approval of an ethics committee is not required by Finnish law or ethical principles ^83^. Researcher need to apply with a detailed specific research plan on planned data linkages to receive a statement from the regional ethics committee within the hospital district when register data is requested to be used. The application for data permission has to include a data utilization plan, a list of each researcher that will process the data, and a data description. An amendment must be submitted if the application is alternated (e.g. adding researchers) ^38, 39^. The DPIA is required prior to processing if data processing is likely to result a high risk to data subjects like processing on large scale of health data ^84^.

### Norway

***Legal basis for research.*** Norway is not member of the EU but a member of the EEA and thus bound by the GDPR in the same manner as EU member states. The Act on the Processing of Personal Data (Personal Data Act) incorporates EU’s GDPR to Norwegian law and contains national rules in areas where the GDPR allows it ^85^. Researcher must comply with both the main rules of the GDPR and the special rules of the Personal Data Act when processing personal data. Other regulations for research using medical data are the Act on Medical and Health Research (Health Research Act) of June 2008 ^86^ (altered by the Act on Amendment in Personal Health Data Filing System Act of January 1, 2021) ^87^, and the Act on Ethics and Integrity in Research (Research Ethics Act) of April 2017 aiming to ensure that research is conducted according to recognised ethical standards ^88^. The acts are further specified by regulations and guidelines.

The regulatory bodies of health research are the Norwegian Board of Health Supervision attending the legal reliability of research ^89^, and the Norwegian Data Protection Authority providing guidance and advice on data protection ^90^.

According to the Health Research Act, the Regional Committees for Medical and Health Research Ethics (REC) has the authority to evaluate whether research projects fulfil the criteria set for medical and health related projects ^85^. The projects must also be compliant with the Personal Data Act ^91^. The research institutions are responsible for establishing guidelines, procedures, and systems to be compliant with all laws relevant for medical and health research. Research projects that process personal data within other fields than medical and health science have duty to report to the Norwegian Centre of Research Data ^92^, a national archive offering help to assess whether research projects meet the requirements of data protection legislation.

***Legal basis for registries.*** Norway holds various national registries storing health related data as well as education and demographic data. The national registries are regulated by the Act on Personal Health Data Filing Systems and the Processing of Personal Health Data (Personal Health Data Filing System Act) of June 2014 to promote health and prevent disease ^93^ (altered by the Act on Amendment in Personal Health Data Filing System Act of January 1, 2021) ^94^, and the Act relating to official statistics and Statistics Norway (Statistics Act) aiming to ensure official high-quality statistics to inform public, research and guide decision-making ^95^. Registries that are not based on consent to file data are for example the Medical Birth Registry Norway, the Norwegian Patient Registry, or demographics filed by Statistics Norway. Furthermore, several medical quality registers are established ^96^, some hold duty to report without consent, such as the Norwegian Cardiovascular Disease registry, while most of them are based on consent, for example the Norwegian Cerebral Palsy Registry.

***Representation of minors.*** According to the Health Research Act §17, the right to consent is generally from 18 years, and from 16 years if research does not involve bodily intervention or testing medical products, in which case the guardian (legal representative) must consent ^97^. According to the regulation and provided that the Regional Ethics Committee approves, minors between 12 and 16 years can themselves consent to research on medical matters if the public utility exceeds the possible disadvantages or if interests may conflict between the child and the parent/guardian (e.g., violence or neglect) ^98^.

***Opportunities to link.*** In Norway every citizen and permanent resident has a unique national 11-digit personal identifier for all registrations which is provided at birth or at immigration and reported confidential but without necessary consent to the national registries as defined by §11 in the Personal Health Data Filing System Act ^99^. The personal identifier can be used to link personal data with register data if REC approves, either based on consent or for well-founded public, scientific, historical, or statistical purposes. The sharing of indirect identifiable individual level data with other countries is possible through strict regulations.

***Record linkage with other databases.*** Statistics Norway administers official statistics about the Norwegian society. This includes data on education, income, social and work-related information. Access to indirect identifiable data for research purposes is regulated by the Statistics act §14 ^100^. Statistics Norway is given the authority to regulate procedures for access to data ^101^. Linking clinical health data with register data is possible, given that the required ethical and legal regulations are fulfilled, and the researchers are affiliated with an approved research institution by either the Research Council of Norway or Eurostat ^102^. An overview of data sources is given by Helsedata ^103^ which includes more than 40 registers with health data, and guidelines are prepared for access to microdata from Statistics Norway ^104^.

The Act on Amendment in the Personal Health Data Filing System Act enforced in January 2021 is aiming to make it easier and safer to make health information available for statistics and research ^105^. The National Health Analysis Platform is a technical platform that will provide researchers with tools to conduct new types of health and medical research. The platform will facilitate complex analysis across the different registries and other relevant sources of health information and improve information security and protection of special data categories.

***Procedural conditions.*** All projects that fall within the jurisdiction of the Health Research Act must according to § 9 and 10 apply for pre-approval to the Ethics Committee in order to start the project ^106^. Informed written consent is a premise for sampling and accessing individual health data. Therefore, the written participant information and consent form, if relevant describing linkage to specified registries and data sharing with other countries, must be approved by the committee. Exemptions from requirements of consent are 1) minimal risk for not ensuring the well-being and integrity of participants, 2) substantial interest for society, and 3) consent is difficult to collect for various reasons, and requiring consent will cause incomplete data set, introduce bias and considerably hamper the research quality. The application must furthermore include: i) a project description with aims and justifications for the need for new knowledge, ii) details on planned data linkages; iii) reasoning on the necessity of using the data for the project; iv) who will have access to data; v) and how data will be stored ^107^. An amendment must be submitted if the original application is changed.

Linking individual clinical data with register data requires approval from an Ethics Committee and accommodation to the Personal Data Act fulfilling EU’s GDPR. Pseudo-anonymised data with low risk for indirect re-identification may be shared with research collaborators nationally and within EU/EEA with a strict control on access to data ^108^. Registry authorities usually secure only few analysts, ideally one analyst for a study. Such human restriction jointly with data minimisation and adequate technical solution safeguard data protection. For lending individual data on education and demographics, an application must be submitted to Statistics Norway for a specific research project and for a specified period. Researcher affiliated with an authorised research institution may apply.

### The Netherlands

***Legal basis for research.*** The national implementation of the GDPR was finalized on March 25, 2018 in the Implementing Data Protection Act called Uitvoeringswet Algemene Verordening Gegevensbescherming ^109^. In addition, several changes have been made to other legislation such as in the Act on the National Institute of Public Health and Environment ^110^. The emphasis in Dutch implementation of the GDPR was first of all not to change the content of existing legislation which was deemed to be compliant with the GDPR. In the second place not to reiterate in Dutch legislation which follows directly from the GDPR already. Hence, the necessity for appointing a DPO or performing a DPIA follows directly from the GDPR and not from Dutch law. The same applies to the principle of data minimisation and other GDPR principles.

Legislation which was not changed involves amongst other the act on the treatment contract, which is part of the Dutch Civil Code and dates from 1995 already ^111^. The treatment contract Act covers various patient rights such as informed consent for treatment, the right to a copy of the medical file and the right to professional secrecy. The Act on medical research with human beings was not changed either because of the GDPR. This Act has a limited scope of application applying only to medical scientific research where the participants are subject to procedures or are required to follow rules of behaviour ^43^. Purely observational research does not follow under the remit of the Act, also when that would involve occasionally filling in questionnaires. Hence, in the context of this paper, this Act will not be discussed.

There can be various legal bases to establish a cohort with research participants. A cohort which recruits volunteers obviously would require their consent and several of these large cohorts exist in the Netherlands, either population based ^112^ or targeting a specific group ^113^. All these cohorts with volunteers are based on broad consent predating the GDPR. Some of those cohorts recruit subgroups for add-on studies which will fall under the remit of the Act on medical research with human subjects when the add-on study involves specific tests or procedures to follow. Such add-on studies there will then be based on specific consent.

Cohorts can also be based on secondary use of health data. While data processing for the provision of health care is not based on consent but on the treatment act which requires the doctor to keep a medical file, in general, the consent of the patient is required to release patient data to a party not involved in the treatment ^114^. There are exceptions. A breach of professional secrecy can be required by law such in the context of the health insurance reimbursement system ^115^ or with notifiable communicable diseases ^116^. There is also an exception for research. Consent is not required when it would be impossible or when it would be unfeasible to ask for consent, the research serves a public interest, the privacy of the data subject is sufficiently assured (in practice meaning that the data should pseudonymised), the research cannot be performed without those data and the patient did not opt-out to such use ^117^. These four conditions release data by the treating physician to a researcher are reflected in somewhat different wording in article 24 (and article 27 for genetic data) in the Dutch implementing Act as the legal base for the research institution to process health data without consent.

This legal base can in general not be used by a cohort with active volunteers. As there is some form of contact, consent can be asked. Therefore, for linking with data from the health care system and registries, consent will be asked. Usually at the start of cohort on the consent form with tick boxes for various databases.

In 2004 the Dutch health research community issues a Code of Conduct on health research which was approved by the existing data protection authority ^44^. That Code of Conduct already had provisions which are now laid down in the GDPR such as that the research protocol should explain how about data minimisation and pseudonymisation of the research data is being applied. The Code of Conduct is at the moment under revision ^118^.

There is substantial discussion whether the existing broad consent cohorts can retain their present procedures and about the relation between the consent to submit data to a researcher for further use for research as follow from the Act on the treatment contract and consent in the sense of the GDPR. Some authors claim that consent according to the treatment contract can be broad but the researchers should still fall back on the exception to the consent principle of article 24 of the Dutch implementing Act ^45^.

***Legal basis for registries.*** Except for a clause in the Act of the National Institute for Health and the Environment relating to not notifiable communicable diseases, the Netherlands has no formal regulation on health registries implementing article 9.2.i GDPR. Some registries are based on the opt-out system discussed above. Other function on the basis of disputable controller-processor basis, the registry then being the processor. Legislation regarding quality registries has been announced by government ^119^. Those quality registries are meant to give feedback to health care providers about their performance on certain quality indicators compared to the average of all participating health care providers. Such quality registries will not be based on consent in order to assure their integrity. It remains to be seen whether the non-anonymised data from these quality registries may also be used for research without consent.

The largest registry or a bundle of registries is held by Statistics Netherlands (SN). SN is based on its Act incorporating the European legislation regarding statistical agencies. As any statistical agency SN require citizens, corporations and public bodies to submit personal data to it. SN has detailed data about for example the education, health and welfare consumption and income of citizens. The Act on Statistics Netherlands contains special provisions on the use of the data for research ^120^. The data of SN can be used for research if certain conditions are met. If the participant has explicitly consented to linking with SN, the data from SN may be added to the cohort data. If there is not such explicit consent, the cohort data may be submitted to SN which will perform the linking. The researcher can then analyse the dataset within the secure environment of SN also by remote access. SN has then procedures that the researcher can only extract the fully anonymous results of the statistical analyses ^121^.

***Representation of minors.*** A distinction should be made here between when the minor is also a patient or at other situations. The Act on the treatment contract gives the minor from 12 years onwards a personal privacy right to the medical record ^122^, hence, to retrieve data from the health record, the child from 12 years onwards, when competent, should decide. For general purposes the age for consent has been set at 16 years in the Implementation Act ^123^.

***Opportunities to link.*** Use of the national registration number is required for many public functions. Health care providers and health insurers are required to use this number ^124^. Data to SN are submitted under the national registration number or a pseudonym which SN can reverse to the national registration number. Within SN the national registration number is then again pseudonymised to the unique SN number.

However, the national registration number may only be used when explicitly allowed by legislation ^125^. There is no legislation which allows that number to be used for research. Hence, linking with SN can never be exact because of wrong spelling etc.

***Record linkage with other databases.*** See above

***Procedural conditions.*** The Act on medical research involving human subjects has a system for accreditation of medical ethical review boards and every protocol falling under the remit of the Act must be approved by such a committee. The Netherlands does not have such a system for observational research. In practice many research institutions have such non formally accredited review boards and so have almost all registries, sometimes named as privacy committees or data access boards. In practice there are no gaps for ethical review but on the contrary because of the lack of a national system there are overlaps where a proposal is reviewed by several bodies, sometimes coming to different conclusions ^46^. There is not a requirement to consult the Data Protection Act (2018) for research unless directly following from the GDPR itself, being that the DPIA would necessitate a consultation of the GDPR. A request for linking data with SN will be reviewed by an access committee unless it has approved a similar request from that research institution already. The procedure is known to be rather quick.

## Discussion

### Summary GDPR application among member states

The GDPR aimed to create a robust and coherent data protection framework across EU/EEA member states by ensuring a constant and high level of protection for the individual and the proper functioning of free movement of personal data within the EU/EEA in order to respond to rapid technological progress, globalization and associated challenges ^30^. The implementation of the GDPR was successful in EU/EEA member states’ encouragement by strengthening the role of data protection authorities and by promoting the allocation of sufficient resources to data protection authorities ^28, 30, 34^. However, the margin that the GDPR allowed for each EU/EEA member state in the national implementation, as demonstrated in the results, appears to have caused for divergence to remain, discouraging innovative research in particular in states with more restrictive implementation ^29, 35, 47, 48^. Fragmentation originating from different approaches followed at member state level seems to have further created unequal settings for researcher challenging data exchange, record linkage, and generally research collaborations within and across EU/EEA member states ^35^.

### Public interest and scientific research

First of all, the principal questions that consider national and cross-national record linkage of cohort data with routinely collected data relate either to the conditions provided for scientific research and public health ^126^ or to the legal basis of public interest ^127^. Even though the GDPR lays down specific requirements for the processing of sensitive data, the GDPR does not automatically signify that data may be processed in agreement with these allowances, as the allowances rely on further EU or national legislation. Neither does the GDPR signify that the legal basis always has to be the provision of an explicit consent as there are several other legal grounds in the GDPR to process personal data ^35^. The GDPR generally prohibits the processing of sensitive data ^128^ (e.g., genetic ^129^, biometric ^130^ and health ^131^) but lifts this prohibition in particular scenarios ^132^ such as for scientific research purposes ^133^ which is subject to the imposed obligations ^134^. Thus, the GDPR acknowledges scientific research as a legitimate purpose for data processing and as a specific condition for the processing of sensitive data ^135^ although requiring further regulation and a legal basis to be used in practice.

A lawful ground of personal and sensitive data processing ^136^ must always be fulfilled by the researcher and/or the research institution, acting as a data processor and/or controller. For instance, data processing is permitted if it is necessary for a task carried out in the public interest ^137^. Data processing is also permitted if essential to attend the legitimate interests of a controller or a third party ^138^, yet, public authorities cannot process data in the performance of their tasks ^139^ relying on legitimate interest. Moreover, the GDPR limits the exemption of data processing for scientific research to those cases where a national or Union Law provision regulates it, in accordance with certain technical and organizational measures ^140^. Hence, research entities cannot solely rely on the research exception ^35^. The GDPR leaves significant room for national (or specific EU) legislation ^141^. In particular, on the one hand, it allows exceptions to the informed consent principle in the context of research which need to be laid down in EU or national member state law ^142^, while, on the other hand, it allows EU/EEA member states to maintain or introduce further conditions including limitations with regard to the processing of genetic, biometric and health data (e.g., explicit consent and written informed consent). The existence of national limitations also conditions the application of the presumption of combability of secondary use of data ^143^. Thus, the GDPR allows substantial national variations and therewith also the possible regulatory fragmentation across EU/EEA member states ^49^.

This fragmentation can be seen in our results: The **Netherlands, Finland** and **Norway and Portugal** have in place some exceptions and derogations from data subjects’ rights also applicable to health data related research, which may apply to record linkage. However, only in Finland a dedicated Act on the Secondary Use of Health and Social Data and research was approved that is typically grounded on public interest and the necessity for scientific research as the main legal basis for the use of health data. In fewer cases consent is used. In all the four countries less restrictive conditions apply to non-sensitive data (e.g. education data), without prejudice to the need to ensure lawfulness of the processing. In **Portugal** – which currently seems to be the most restrictive of the four assessed countries—if sensitive or non-sensitive data is anonymized, and therefore cannot be linked to the data subject, its access can be granted for research purposes ^144^; otherwise, an explicit or an explicit and written consent (the latter in the case of routinely collected data held by the health system) must be given ^145^ and can only be disregarded in very exceptional circumstances. Yet, particular rights of the data subjects can be derogated in the context of scientific research, subject to certain conditions, when they are likely to render impossible of seriously impairing the objectives of the research in question. Nevertheless, anonymized data falls outside of the scope of the GDPR and member states' personal data legislation, as the GDPR does not apply for anonymized data ^146^. Thus, even though room for national legislation is granted ^147^ which to some extent is beneficial, it also implies a potential risk of regulatory fragmentation ^148^ ^35, 49^. Even though the GDPR aimed to avoid regulatory fragmentation across EU/EEA member states, any national derogations allowed by the GDPR ^149^ similarly upsurges this possibility ^35, 49^. Thus, we argue that EU national legislatures ought to further collaborate and work jointly together to guarantee consistency ^35, 49^. There is also room for further EU legislation in specific matters, as it is the case of the proposed creation of a Health Data Space in Europe for the improvement of data sharing for scientific research purposes ^50, 51^.

### Form and scope of consent

The processing of personal data or special categories of personal data, such as health data, requires the application of the GDPR, as per definition, record linkage is a processing operation which entails higher risks for privacy ^52^. The GDPR establishes several legal grounds to process data of which one of them must be fulfilled, except if data is anonymized and then the GDPR is not applicable ^150^. In practice, regarding sensitive data such as health data: i) either an explicit informed consent ^151^ must be provided in oral or written form, ii) or the basis is a task carried out in public interest (in this case either for reasons of public health ^152^) iii) or because it is necessary for scientific, historical, and statistical purposes ^153^. Albeit one legitimate basis for sensitive data processing is consent ^154^, the GDPR acknowledges that it may not always be possible to fully identify the purpose of personal data processing for scientific research purposes at the time of data collection ^155^. The GDPR even states that it should be allowed to give consent to certain areas of scientific research. Hence, on the one hand, the GDPR places a *normative weight* on the consent as a requirement which deviates in light of each health research setting. On the other hand, the GDPR places a more *substantive approach* to consent as it allows research as an exemption ^53^. Thus, in line with Dove and Chen (2020) the question arises: Should consent for data processing be privileged in health research as a lawful basis? ^54^.

It appears that there is some political and regulatory divergence emerging from this normative connection that is made between consent as a lawful basis in data protection for the data subject and consent as a research ethics principle ^54^. We argue in accord with the European Data Protection Board which inter alia commented, that there are persuasive motives why consent for data processing in the context of health research may not be the suitable lawful basis (reliant also on the kind of project) ^54^. This could be seen in the example of **Portugal**, a member state that is more determined to the value of informational self-determination. In cases where cohort data collected, based on consent to participate in a study, is linked with routine data, we argue that consent can be used as one lawful basis signifying respect for the data subject and balancing the communication with the data controller but to the extent that it does not hamper research practices ^54^. Thus, stronger emphasis should be placed on the purpose of public interest and the scientific research exemption while not undermining data protection and data privacy.

In line with Donnelly and McDonagh (2019), we claim that the GDPR articulates research exemption at a more principled and theoretical level, hence, in praxis the research balance is *struck* at national member state level ^53^. Consequently, the GDPR not only allows complications and barriers for EU/EEA cross-national record linkage and scientific EU/EEA research projects to remain, it allows to hamper its own aim: to create a harmonised regulatory framework for health research ^53^. Moreover, the fundamental values of the existing legislation in each EU/EEA member state in terms of the equilibrium between individual rights to informational self-determination and the common public good can most likely explain this diversity together with infrastructural constraints. Striking in this regard is also the impact of culture on the concept of patient autonomy and informed consent ^55^; therewith, researchers' responsiveness and sensitiveness to cultural differences in national or cross-national studies are key factors in improving study participation and retention and ultimately the quality of research ^56^.

The form and scope of consent to access personal data from registries and to undergo record linkage for research purposes varies greatly across member states. In health research, **Portuga**l requires explicit (mostly written) informed consent ^156^, yet, allowing consent given for areas of scientific research. **Norway** requires ethical approval, which demands well-founded research grounds and preferably that linking is explicitly described in the written background information of the consent form (informed consent). **Finland** mainly requires public and legitimate interest for scientific research and less frequent explicit informed consent. The **Netherlands** require general informed consent with “tick boxes” relying on the opt-out system. Yet strikingly, it could be argued that the GDPR may be more restrictive than any of the member states compared, due to the principle of accountability, which establishes that responsibility must be taken for what is being done with personal data and how other principles are complied with. As appropriate measures and records are needed to validate the compliance, oral consent may rather be seen as a remote scenario as the necessity to record oral consent exists according to the principle of accountability ^157^.

Despite acknowledging that the opt-in consent is a crucial part of patient-centred approach in research for those patients who generally do not opt to participate in research ^7^, we argue that the opt-out approach — as practiced in the **Netherlands** —is a suitable mean of obtaining consent in medical health research and may facilitate record linkage when based on the data protection and privacy rights of the data subjects as well as may encourage research participation ^57, 58^. This should be a factor to consider when aiming to circumvent the growing phenomena of refusals to participate in epidemiological studies ^59– 63^. Nevertheless, upcoming opt-out systems should have a focus on monitoring register performances and the purpose and criteria for evaluation must be determined before the execution ^64^. Yet, with regards to the national implementation of the GDPR, it appears that in the **Netherlands** a tendency to curtail the application of the opt-out system and replace it by generic consent at the start of the treatment emerged. Hence, in the **Netherlands** the debate now hinges around the question whether such a generic consent is compliant with the notion of explicit consent in the GDPR ^158^. The restrictive interpretation of the European Data Protection Board of Recital 33 should then be abandoned, and more emphasis given on how the European Data Protection Supervisor (2020) saw the potentialities of this Recital. Interestingly, also in Portugal the implementation legislation adopted generic consent in line with Recital 33 wording. We recommend aligned to Donnelly and McDonagh (2019), that the European Data Protection Board should offer explicit direction on the process of consent in health research in order to tackle limited research balance at national member states level ^53^.

### Representations of minors and the age of consent

The maturity of minors has been highly discussed resulting in deviating opinions and henceforth different implementations and practices across the EU/EEA exist ^27, 55, 65, 66^. The GDPR provides that consent for the processing of a child's personal data, in relation to Information Society Services, can be given from the age of 16 years onwards and that the holder of parental responsibility must give authorization under this age ^159^. Even though in the majority of the countries consent is qualified from 16 years onwards for most types of health research and regardless of the research topic with 18 years, the assessed countries vary with regards to the representations of minors and the age of consent. **Finland** (15 years) ^160^, **Norway** (16 years) ^161– 163^ and the **Netherlands** (16 years) ^164^ are closer to GDPR’s proposal regarding Information Society Services, and seem to be more liberal in involving and allowing minors in consent provision. **Portugal** (18 years, by default) ^165^ stood out to be most restrictive or protective with regards to the legal age, even though allowing for a case-by-case assessment of maturity and requiring the assent regardless of minors’ age. It means that the Data Protection Law establishes that, offering Information Society Services directly to a child, personal data processing of the data from a child based on consent can be lawfully conducted if the child is at least 13 years old, which is the lowest permitted by the GDPR ^166^. We argue, in line with GDPR’s provision to guarantee that children must understand any information provided to them ^167^, that the legal age of 18 years – as in Portugal—could be lowered to 16 years providing an opportunity to include mature minors and extend their autonomy ^67, 68^. The age of maturity could be scientifically determined ^65, 69^.

### Scopes of intervention (ethical approval)

In **Portugal** ethical approval from an Ethics Committee must be retrieved before the commencement of research ^168^. In **Finland** no ethical approval by an Ethics Committee is needed for the pure registry data-based research or if principle of informed consent is not deviated for non-medical research; however, cohort studies that collect data from participants do require consent. In **Norway,** before the initialization of medical and health related research, written participant information and consent forms must be approved by a Regional Ethics Committee ^169^, and ensuring that the health research is conducted according to ethical standards, including risk-benefit assessment and ethical grounds for data sampling, linkage, sharing with other institutions nationally and internationally. In the **Netherlands,** even though there is no legislation demanding an ethics committee except for scientific health research which includes procedures or requires to follow rules of behaviour, in practice all major research organisations and data holders of databases which can be used for medical research have such a committee which — in the case of multi centre research— do not always reach the same conclusions. Yet, in all four countries assessed different scopes of intervention, DPIA or Ethics Committees, apply.

Whereas **Portugal** appears to be more segmented requiring the approval for research by local, regional or national Ethics Committees depending on the case, **Finland**, the **Netherlands,** and **Norway** appear to be either more centralized so that additional ethical approval is not always needed ( **Finland**), by having one recipient with the authority to approve health research involving collaboration with other institutions ( **Norway**), or by not having any legislation in first place that demands an Ethics Committee ( **Netherlands**). The paradoxical effect of the seemingly lenient Dutch regime is that researchers have to address various data holders and navigate through various committees if they want to combine data for research. There is not one authority which can state that the research is scientifically valid, ethically warranted and compliant with data protection legislation and hence that the relevant data may be opened up for research. We hence argue that a more uniform process with one recipient having the authority to approve the research so that it is not necessary to send a number of various applications in order to start a research project, as it is the case in **Finland**, may be beneficial for research.

EU-funded projects have commonly opted to not construct a central patient-level database; but instead to store data locally, in view of the data protection and privacy regulations in each EU/EEA member state ^70, 71^. Thus, in cross-national projects and multicentre studies involving multiple European institutions from EU/EEA member states, investigators must separately apply to individual Ethical Committees ^72^. As initial data analyses are mainly locally executed alike within-country analyses, cross-national analyses are centrally carried out in compliance with a shared analysis plan on the aggregated results of the other countries ^70, 73^. However, in some EU/EEA countries current ethical approval processes rather prolong and delay research commencement possibly to an unnecessary extend as well as produce challenges in collecting and extracting data from multiple diverse sources; thus, rather impede national and cross-national record linkage processes ^70, 74^. De Lange *et al.*, (2019) concluded that huge variation across Europe in obtaining ethical permission for a non-interventional observational study in Europe exists in the time between application and first approval: 7 days in the **Netherlands**, 50 days in **Norway** and 300 days in **Portugal** ^72^. In line with that, international studies recommend national harmonization on ethical, privacy and institutional review for multicentre trials or multicentre studies ^75, 76^. Correspondingly, Dove and Garattini (2018) concluded in their qualitative study, that numerous experts that have been interviewed recommended several changes to the present ethics review regime for international research in order to diminish inefficiency and inconsistency ^74^. We argue that the current segmentation of ethical committees and approval processes in some EU/EEA countries may rather hinder the incorporation of data subjects and possibly have an adverse effect on external validity ^72^. In line with de Lange *et al.*, (2019) in order to promote research, further harmonization between EU/EEA countries in obtaining ethical clearance for observational and non-interventional studies and registries is required ^72^. We opt that a more uniform process to improve ethical guidance should be followed across EU/EEA member states. Moreover, European projects should thrive for aiming to produce a centralized and harmonized electronic database of cohort data to facilitate record linkage and data exchange across EEA member states ^77^.

### Legal basis for research and registries

The legal basis for research and for registries varies in number, completeness, and accessibility across the assessed countries. The Nordic countries **Finland** and **Norway** appear to be the least restrictive countries which very closely follow the GDPR in their national implementations. **Finland** deviates the least as its legal basis for scientific research mainly requires public and legitimate interest for scientific research and less frequent consent and legal obligations ^170^. In **Norway,** linkage between various registers is possible but strictly regulated ^171^: While some registries or demographics registered by Statistics Norway, as well as some medical quality registers are not based on consent, others demand consent to allow filing health data. In these registries various health and social information are filed and linked to the unique identification number, making register data accurate and robust ^172^. In **Portugal**, the national implementation of the GDPR permits data processing necessary for the creation of centralized health data bases or registries for specific purposes under information security requirements and based on a unique platform. The National Institute of Statistics is allowed by law to carry out the processing of personal data, including sensitive data, and data linkage, namely with other statistical authorities and disease registries sharing anonymized data; yet the collection of sensitive data typically relies on the authorization of data subjects. In the **Netherlands**, the legal basis for registries is based on the opt-out system and research is built on a generic informed consent, the opt-out system, and (newly with the GDPR) a DPIA and the involvement of a DPO. However, if consent is not obtainable, data can be used for scientific research if serving the public interest and assuring the privacy of data subjects through pseudonymization. Yet, even though citizens, corporations and public bodies are required to submit personal data to Statistics Netherlands, no legislation exists which allows the usage if national registration numbers for research in the **Netherlands**.

Thus, whereas some legislations in EU/EEA member states do not allow the usage of unique identification numbers for health research, as the **Netherlands** and **Portugal**, in **Finland** the linking of unique identification numbers for research without explicit consent for the majority of register-based research is allowed. In **Norway** filing information on identification number is routine for many registries but linking individual clinical information with these register data requires ethical approval, i.e., generally that the subject gives consent, although with some exemptions. Noteworthy, even though several EU/EEA member states attain ethical approval and introduce a DPIA complying with the GDPR, registry holders may set different requirements which could be harmonised ^8, 78, 79^. We further argue, based on the example of **Norway** and **Finland,** that linking unique personal identification numbers across registries and with cohort data for research is a strength and should be further explored. Those identifiers are vital for the operations of national healthcare systems which require to uniquely identify an individual across multiple organizations in order to function properly ^80^. Furthermore, those unique identifiers embrace the possibility to link research data, expand data available for individuals, encourage to detect overlap between data collections, and simplify reproduction of research results ^81^.

National Identity Schemes are compound sociotechnical arrangements in which numerous necessities from various stakeholders must be balanced and based on appropriate levels of privacy and security ^82^. The protection of data subjects’ identity is possible when linking those identifiers, if the data curator allocates random identifiers to survey entities but has no access to the data prohibiting identification of respondents ^80^. Another possible option is the ‘Privacy-Preserving Record Linkage’ technique aiming to link data records without revealing concrete personal identifying attributes and adhering to data privacy ^83^ and federated learning. Even though the GDPR has achieved success for digital health, we recommend the strengthening of countries with the national health identifier system and further security for the protection of personal health information which requires political determination and alliance among all involved stakeholders to function effectively ^84, 85^.

### Record linkage and harmonization

The comparison of the possibilities in linking routinely collected health and education data with cohort data enables to lawfully understand the barriers, challenges, and opportunities across the EU/EEA region. In **Portugal,** if the basis of the data processing reasoning is scientific research, it is possible to link routinely collected health and education data of children with cohort data if data processing has a lawful ground, follows the rights of data subjects, duties of processors and controllers, and the legal requirements and obligations relative to DPIA. In **Finland**, the centralized authority FinData permits the processing of social and health data for scientific research if data controllers – registers of multiple public data controllers, single private data controller (Ombudsman) or the Finnish National Institute of Health – have transferred the permit to FinData ^173^. The Data Permit Authority FinData has the right to request a data permit application statement from the data protection supervisory authority Ombudsman, which was mandatory in the previous law prior to the GDPR. However, this statement is not used for record linkage, but for the general permit of data and is in practice hardly ever requested ^174^. **Norway** allows linking routinely collected cohort data if the Regional Committee for Medical and Research Ethics approved it, and if it complies with the Personal data Protection Act and the GDPR procedures. In the **Netherlands**, linking routinely collected data at statistics Netherlands with cohort data is possible with a generic informed consent based on the opt-out system and the requirement of the conduction of the DPIA and the appointment of a DPO.

Thus, did the GDPR recognize the importance of health research ^31^? The analysis of the four countries revealed that fragmentation and divergence remained, which is not always in favour for linking routinely collected health and education data with cohort data purposing scientific research. One the one side of the spectrum, **Portugal** stood out as the country with the most incomplete national implementation of the GDPR with regards to scientific research compared to any of the other member states assessed ^28^; in part for that reason and the resulting lack of legal certainty it is also considered the most restrictive one, including in what concerns internal and transnational record linkage. Further legislation or the revision of the existing legislation applicable to health-related research would definitely be welcome for the sake of clarity and legal certainty. On the other side of the spectrum, **Finland** appears to be the least restrictive in facilitating record linkage, which amplified the usage of electronic registries comprising sensitive data while maintaining citizen's right to privacy as health care authorities have the right to collect and record health data of individual citizens ^8^. It appears that the GDPR rather has brought a more narrowed and restrictive focus in those EU/EEA member states who had followed a more liberal approach up until now, such as the **Finnish** legislation on the processing of health data and the **Dutch** legislation which now requires additionally the conduction of a DPIA and the consultation of a DPO.

We argue in line with Sorbie *et al.* (2021) that even though data ownership and the idea of ‘my data’ is central for notions of reward, opportunity and control– as executed in **Portugal** –, ethical and social concerns of data that reinforce biomedical research are of greater importance ^86^. Thus, based on the **Finnish** example, a centralized management of national data may be beneficial for research outputs serving as a powerful basis of data at national level and will further facilitate cross-national record linkage of data. Moreover, member states that are more committed to the value of informational self-determination, such as **Portugal,** are rather hindered in conducting health research purposing to contribute to the public good. Even as the GDPR may appear discouraging for researcher in some member states, as Cornock (2018) argues, it rather essentially gives existing best ethical practice a legal standing ^87^. Hence, further consistency and harmonization would be beneficial in line with the response of the European Data Protection Board to the European Commission on the subject of health-related research and the GDPR ^88^. We argue in line with the European Data Protection Board, which recommends that the European Commission should explore the possibility of providing a uniform regime for health-related research in a future legislative proposal dedicated to the European Health Data Space ^50, 51^.

Thus, consistent with Townend (2018) we ask: “Is harmonization an impossible dream” ^89^, or actually practically achievable? And if achievable, how can the impossible be made possible? Townend (2018) further argues that harmonization would be possible if the aim of data sharing – and therewith subsequent record linkage processes – is based on public interest, social liberalism as a basis of solidarity, with an understanding of the human rights approach and citizen sensitivities acknowledging the profession of ‘researcher’ ^89^. Moreover, in line with van Veen (2018), ‘good research governance’ can enable to frontward on consent based research – as in Portugal with individual informational self-determination – or anonymization ^29^. Hence, harmonization may be achievable while taking the opportunities of the flexibilities of the GDPR into account without undermining data protection and data privacy of data subjects. Scientific research that involves sensitive data should be planned to accommodate the needs of the public good considering that personal data protection is not an absolute right ^175^. The protection of personal data should be considered with regards to its purpose in society and in relation to the principle of proportionality ^176^ and be balanced with other fundamental rights ^90^, as otherwise harmonization across EU/EEA member states is hampered in disadvantage to record linkage purposing research.

### Strengths and limitations

This comparison can assist researchers aiming to establish international collaboration with other countries and help to handle with the technical aspects of the data transfer/processing etc. The comparison allowed to understand the best practices for research from each EU/EEA member state. The study is limited to a lawful view on record linkage.

## Conclusion

Even though the GDPR is the most important legal framework for the protection of personal data in Europe, the national execution, when it concerns registries and research, matters most for record linkage. However, this varies: where in some EU/EEA states registers with which one could theoretically link data do not even exist while in other member states the registers exist and linking is possible without explicit consent. Underlying values of the existing legislation in each member states, concerning the balance between the individual right to informational self-determination and the public good can most probably explain that diversity along with infrastructural limitations and also the pace and completeness or sufficiency of the GDPR implementation reforms. Researchers from member states more committed to the value of informational self-determination, such as Portugal, are often hampered in doing research which in their opinion would contribute to the public good. It will remain a challenge to overcome these variances in Europe. More harmonization could be helpful but should certainly not be detrimental for research in those member states which opened a leeway for registries and research for the public good without explicit consent.

## Data availability

All data underlying the results are available as part of the article and no additional source data are required.
