# Supplementary material for: Record linkage of population-based cohort data from minors with national register data: a scoping review and comparative legal analysis of four European countries
Source: Open Res Eur. 2021 Sep 27;1:58. Originally published 2021 May 27. [Version 2] doi: 10.12688/openreseurope.13689.2 (PMC10445839; doi:10.12688/openreseurope.13689.2)
Supplement: Peer review history

# Peer review: Reviewer response for version 2

**DOI:** 10.21956/openreseurope.15260.r27688

**Published:** 2021-11-26

**Authors:** Mark Taylor

**Affiliations:** 1 Melbourne Law School, Melbourne University, Melbourne, Australia

I’ve reviewed and think the changes made are adequate.

Are the rationale for, and objectives of, the Systematic Review clearly stated?

Yes

Is the statistical analysis and its interpretation appropriate?

Not applicable

Are sufficient details of the methods and analysis provided to allow replication by others?

Partly

Are the conclusions drawn adequately supported by the results presented in the review?

Partly

Reviewer Expertise:

NA

I confirm that I have read this submission and believe that I have an appropriate level of expertise to confirm that it is of an acceptable scientific standard.

# Peer review: Reviewer response for version 2

**DOI:** 10.21956/openreseurope.15260.r27690

**Published:** 2021-10-11

**Authors:** Mahsa Shabani

**Affiliations:** 1 Metamedica, Faculty of Law and Criminology, Ghent University, Gent, Belgium

I have reviewed the responses of the authors and I  have no further comments on the manuscript.”

Are the rationale for, and objectives of, the Systematic Review clearly stated?

Partly

Is the statistical analysis and its interpretation appropriate?

Not applicable

Are sufficient details of the methods and analysis provided to allow replication by others?

Partly

Are the conclusions drawn adequately supported by the results presented in the review?

Partly

Reviewer Expertise:

NA

I confirm that I have read this submission and believe that I have an appropriate level of expertise to confirm that it is of an acceptable scientific standard.

# Peer review: Reviewer response for version 1

**DOI:** 10.21956/openreseurope.14763.r27015

**Published:** 2021-08-03

**Authors:** Anders Foldspang

**Affiliations:** 1 Department of Public Health, Aarhus University, Aarhus, Denmark

Doetsch *et al*. present a thorough, detailed, systematic, legal, comparative analysis and scoping review with the objective to investigate possibilities of linking cohort data of minors (as an example) with routinely collected education and health data, comparing EU/EEA member states, with a central view to the implementation of the General Data Protection Regulation (GDPR) in Portugal, Finland, Norway, and the Netherlands.

 Use of registers and data linkage is crucial for the production of knowledge in public health science and practice, including epidemiological population studies as well as studies on health services functioning and studies in clinical epidemiology. Linkage of demographic data with health, social and economic data opens up hitherto non-accessible scientific and practical perspectives – and not least when the complicated phenomenon of cross-border linkage is made possible.

 Having considered the special conditions of the implementation of the GDPR in each of the four countries selected, Doetsch *et al*. discuss public interest and scientific research; form and scope of consent; representation of minors and the age of consent; scopes of intervention and ethical approval; the legal basis for research and registries; record linkage and harmonization. 

 These months, the global Covid-19 pandemic has demonstrated both the use of and the need for high-quality population data – not tomorrow but in time – from all European states and from states outside Europe as well. Register-based information has been crucial to the initiation and implementation of large and extremely expensive anti-Covid-19 programmes. Thus, on one side, removal of obstacles to scientific and practical register use necessarily must be extremely precise and of high quality, whereas it, on the other side, as it seems, can only happen too slowly.

 Thus, the detailed problem analysis and discussion presented by Doetsch *et al*. is future-oriented and a gift to the process of development of register refinement and coverage as well as register and data linkage and analysis in research and practice. The Covid-19 pandemic has demonstrated the urgent need for that.

Are the rationale for, and objectives of, the Systematic Review clearly stated?

Yes

Is the statistical analysis and its interpretation appropriate?

Not applicable

Are sufficient details of the methods and analysis provided to allow replication by others?

Yes

Are the conclusions drawn adequately supported by the results presented in the review?

Yes

Reviewer Expertise:

NA

I confirm that I have read this submission and believe that I have an appropriate level of expertise to confirm that it is of an acceptable scientific standard.

# Response

**Published:** 2021-09-17

**Authors:** Julia Nadine Doetsch

**Affiliations:** EPIUnit- Instituto de Saúde Pública da Universidade do Porto (ISPUP), Portugal

**The authors' responses to the reviewer report 1 were highlighted in bold.**

 Doetsch *et al*. present a thorough, detailed, systematic, legal, comparative analysis and scoping review with the objective to investigate possibilities of linking cohort data of minors (as an example) with routinely collected education and health data, comparing EU/EEA member states, with a central view to the implementation of the General Data Protection Regulation (GDPR) in Portugal, Finland, Norway, and the Netherlands.

 Use of registers and data linkage is crucial for the production of knowledge in public health science and practice, including epidemiological population studies as well as studies on health services functioning and studies in clinical epidemiology. Linkage of demographic data with health, social and economic data opens up hitherto non-accessible scientific and practical perspectives – and not least when the complicated phenomenon of cross-border linkage is made possible.

 Having considered the special conditions of the implementation of the GDPR in each of the four countries selected, Doetsch *et al*. discuss public interest and scientific research; form and scope of consent; representation of minors and the age of consent; scopes of intervention and ethical approval; the legal basis for research and registries; record linkage and harmonization. 

 These months, the global COVID-19 pandemic has demonstrated both the use of and the need for high-quality population data – not tomorrow but in time – from all European states and from states outside Europe as well. Register-based information has been crucial to the initiation and implementation of large and extremely expensive anti-COVID-19 programmes. Thus, on one side, removal of obstacles to scientific and practical register use necessarily must be extremely precise and of high quality, whereas it, on the other side, as it seems, can only happen too slowly.

 Thus, the detailed problem analysis and discussion presented by Doetsch *et al*. is future-oriented and a gift to the process of development of register refinement and coverage as well as register and data linkage and analysis in research and practice. The Covid-19 pandemic has demonstrated the urgent need for that.

**Authors' reply: We would like to thank the reviewer Prof. Anders Foldspang for their constructive comment and positive response.**

# Peer review: Reviewer response for version 1

**DOI:** 10.21956/openreseurope.14763.r27098

**Published:** 2021-07-19

**Authors:** Mahsa Shabani, Pieter De Smet

**Affiliations:** 1 Metamedica, Faculty of Law and Criminology, Ghent University, Gent, Belgium; 2 Ghent University, Ghent, Belgium

The manuscript uses scoping review methodology to review the relevant national regulations in four countries related to linkage of registry databases and cohort data (including education and health data) from minors.

 Below please see our remarks on the version 1 of the manuscript.

 A general remark regarding the writing style/wording choices of the manuscript: attention should be paid to the way that sentences are written. Below I list some examples that require revisions:

- From abstract:  "Linkage of data directly collected from cohort participants based on individual consent must respect data protection rules and privacy rights of data subjects." In principle, in processing any type of personal data, regardless of the legal basis for data collection (consent or others), data protection rules and rights must be respected.
- Another example from the abstract: “EU/EEA has limited legislative authority over member states.” This is a strange phrase, as EU law is generally considered to have primacy over national law (depending on the subject matter).
- Another example: P. 4: e-privacy regulation: the regulation is not yet approved by the relevant institutions – the replacement of the Directive is thus not (yet) definite.
- Example from P. 21: the legal basis for scientific research is also a public interest basis, so the comparison/listing is not fully accurate.
- example from P. 16: It’s not because Norway forms part of the EEA, that Regulations/Directive of the EU evidently apply. This requires an agreement within EEA/EFTA and an additional protocol – would be interesting to link to that agreement (cfr/ https://www.efta.int/EEA/news/General-Data-Protection-Regulation-incorporated-EEA-Agreement-509291).
- This sentence is not correctly written: "As a result, the so called trialogue between the European Parliament, the Council and the European Commission as substantial leeway was left to the member states".
- In table 3, under column "GDPR specific articles" also recitals have been included. this is not fully accurate as in principle recitals are not articles.
- Related to this general remark, the language errors and typos in the text need to be fully addressed. 

Here are other comments on the content of the paper: 

- Abstract: The abstract is not fully informative. It would be better that to start why this type of data processing is of interest to this study (maybe by reference to the project).
-  In the methods section, it should be specified that the search string has been adopted based on the local languages. Also, it would be helpful to include whether the search has been conducted and checked by multiple researchers and how this has been coordinated among various authors.
- In the methods, the review question is quite broad and vague ( "what are the possibilities of..."). It is important to nuance this and make it more specific. Otherwise, this can also refer to technical possibilities, etc.

Results and discussion: 

- In general, the rationale behind table 3 is not very clear to me. How columns and rows are related to each other? Or this is in principle a table with standalone columns?
- A general remark: in the paper, approvals and requirements from data protection regulations and research with human subjects (ethics approvals for processing health data mainly) have been discussed together, while this has not been adequately explained in the introductory part. For a reader with no prior knowledge on this matter, this lack of distinction can be confusing.
- In the paper has been referred to binary option of annonymisation vs consent as legal basis. However, it has not been adequately explained what are the potential risks associated with re-identifiability of data in this context? is this in principle possible?
- Use of privacy preserving methods has been briefly mentioned in the manuscript. what are the other technical and organisational measures that can/must be used in this context in case data have been processed based on research exemption rules?
- Page 21 out of 27 (under form and scope of consent): The lawful grounds for processing sensitive data is broader than what is listed there (although these might be the most relevant ones for this discussion). In any case, to avoid confusion, it is important that nuance this paragraph.
- The authors mention that the upcoming European Health Data Spaces can be a potential solution to address the current fragmented regulatory framework in the EU regarding secondary uses of data. It would be interesting to briefly elaborate on that and discuss how this can address the current regulatory fragmentation in the EU on this matter.

Are the rationale for, and objectives of, the Systematic Review clearly stated?

Partly

Is the statistical analysis and its interpretation appropriate?

Not applicable

Are sufficient details of the methods and analysis provided to allow replication by others?

Partly

Are the conclusions drawn adequately supported by the results presented in the review?

Partly

Reviewer Expertise:

My expertise in in the area of health privacy, with focus on processing health data for scientific research purposes.

We confirm that we have read this submission and believe that we have an appropriate level of expertise to confirm that it is of an acceptable scientific standard, however we have significant reservations, as outlined above.

## References

[1] : General Data Protection Regulation incorporated into the EEA Agreement. Reference source

# Response

**Published:** 2021-09-17

**Authors:** Julia Nadine Doetsch

**Affiliations:** EPIUnit- Instituto de Saúde Pública da Universidade do Porto (ISPUP), Portugal

**The authors' responses to the reviewer report 1 were highlighted in bold.** 

 The manuscript uses scoping review methodology to review the relevant national regulations in four countries related to linkage of registry databases and cohort data (including education and health data) from minors.

 Below please see our remarks on the version 1 of the manuscript.

**Authors' reply: We would like to thank the reviewers Prof. Mahsa Shabani and Dr. Pieter De Smet for their thorough revision and detailed comments.**

 A general remark regarding the writing style/wording choices of the manuscript: attention should be paid to the way that sentences are written. Below I list some examples that require revisions:

- From abstract: "Linkage of data directly collected from cohort participants based on individual consent must respect data protection rules and privacy rights of data subjects." In principle, in processing any type of personal data, regardless of the legal basis for data collection (consent or others), data protection rules and rights must be respected.

**Authors' reply: That is correct, data processing of any type of personal data, regardless of the legal basis for data collection (consent or others), must respect the data protection rules and rights. As we focus on this particular scenario, we narrowed it down in the text to data directly collected from cohort participants. We cut out the “based on individual consent” to avoid misunderstandings, as here we referred to ethical approval.**

- Another example from the abstract: “EU/EEA has limited legislative authority over member states.” This is a strange phrase, as EU law is generally considered to have primacy over national law (depending on the subject matter).

**Authors' reply: Despite being a Regulation (directly applicable), the GDPR does not ensure total uniformity in data protection legislation across member states. It contains a significant number of provisions either permitting or requiring Member States to make their own national implementation in specific fields. As a result, national variations remain in some areas.” We adapted the sentence to make it clearer. Another example: P. 4: e-privacy regulation: the regulation is not yet approved by the relevant institutions – the replacement of the Directive is thus not (yet) definite. Authors' reply: We agree the way it was written may have led to potential misunderstandings. We adapted the sentence to: "The GDPR along with the e-privacy directive, covering electronical communication to be replaced by the e-privacy regulation, functions as the ultimate legal framework on data protection and data privacy that reinforces individual control of data subjects’ own data and their associated rights in a digitalized era.**

- Example from P. 21: the legal basis for scientific research is also a public interest basis, so the comparison/listing is not fully accurate.

**Authors' reply: We adapted the enumeration to be more precise.**

- Example from P. 16: It’s not because Norway forms part of the EEA, that Regulations/Directive of the EU evidently apply. This requires an agreement within EEA/EFTA and an additional protocol – would be interesting to link to that agreement (cfr/ https://www.efta.int/EEA/news/General-Data-Protection-Regulation-incorporated-EEA-Agreement-509291).

**Authors' reply: Thank you, we adapted the sentence to make it clearer. We also included this explanation in the footnotes.**

- This sentence is not correctly written: "As a result, the so called trialogue between the European Parliament, the Council and the European Commission as substantial leeway was left to the member states".

**Authors' reply: We adapted the sentence to: Thus, the result of the so called trialogue between the European Parliament, the Council and the European Commission left a substantial leeway to the member states in its implementation".**

- In table 3, under column "GDPR specific articles" also recitals have been included. this is not fully accurate as in principle recitals are not articles.

**Authors' reply: We agree that the headline should be more inclusive and adapted the titles.**

- Related to this general remark, the language errors and typos in the text need to be fully addressed. 

**Authors' reply: We proofread the manuscript again for typos.**

 Here are other comments on the content of the paper: 

- Abstract: The abstract is not fully informative. It would be better that to start why this type of data processing is of interest to this study (maybe by reference to the project).

**Authors' reply: We adapted the second sentence to highlight this study’s interest: "Linkage of data directly collected from cohort participants, potentially serving as a prominent tool for health research, must respect data protection rules and privacy rights.”**

 In the methods section, it should be specified that the search string has been adopted based on the local languages. Also, it would be helpful to include whether the search has been conducted and checked by multiple researchers and how this has been coordinated among various authors. Authors' reply: We added to the methods that the search string has been adopted based on the local languages (see page 5, paragraph “Search”). We also added that the search was furthermore checked by involved researchers in their respective country of expertise. In the methods, the review question is quite broad and vague ( "what are the possibilities of..."). It is important to nuance this and make it more specific. Otherwise, this can also refer to technical possibilities, etc.

**Authors' reply: The research question was on purpose held broad to cover all sub scenarios involved. We adapted the research question to “law possibilities” to make it more distinct that we discuss law scenarios from a legal perspective.**

 Results and discussion: 

- In general, the rationale behind table 3 is not very clear to me. How columns and rows are related to each other? Or this is in principle a table with standalone columns?

**Authors' reply: Yes, table 3 is a table with standalone columns. The same applies for table 1. The format of the tables was determined by the editorial staff of the journal after submission. We asked to strengthen the vertical lines to highlight that the columns are standalone.**

- A general remark: in the paper, approvals and requirements from data protection regulations and research with human subjects (ethics approvals for processing health data mainly) have been discussed together, while this has not been adequately explained in the introductory part. For a reader with no prior knowledge on this matter, this lack of distinction can be confusing.

**Authors' reply: Thank you for this remark. To not cause confusion we did not explore this into depth in the introduction. Later on in the main text, we differentiate in the country specific sections. We added an explanatory sentence in the end of the paragraph to make it clear in the introduction: "Also applicable ethically informed legal requirements vary from country to country."**

- In the paper has been referred to binary option of anonymisation vs consent as legal basis. However, it has not been adequately explained what are the potential risks associated with re-identifiability of data in this context? is this in principle possible?

**Authors' reply: This is a very interesting thought. As the article refers to the possibilities of linking data, this is unfortunately out of the real scope of the article. We did not cover this aspect into depth as it is also not possible to explain this in a concise way and another article on this specific topic would be needed. The identification of risks itself is part of the privacy assessment which we mentioned in several occasions as one of the applicable legal requirements.**

- Use of privacy preserving methods has been briefly mentioned in the manuscript. what are the other technical and organisational measures that can/must be used in this context in case data have been processed based on research exemption rules?

**Authors' reply: The privacy preserving methods is just one of multiple examples. There are plenty of other methods but that would be the subject of another article. The federated learning technique that was mentioned, is practiced in the RECAP preterm project through data analysis in a federated database, which has inspired the motive for this article. We added a footnote.**

- Page 21 out of 27 (under form and scope of consent): The lawful grounds for processing sensitive data is broader than what is listed there (although these might be the most relevant ones for this discussion). In any case, to avoid confusion, it is important that nuance this paragraph.

**Authors' reply: Thank you. Yes, indeed the ones listed were the most relevant ones for the discussion. We adapted the text to avoid confusion.**

- The authors mention that the upcoming European Health Data Spaces can be a potential solution to address the current fragmented regulatory framework in the EU regarding secondary uses of data. It would be interesting to briefly elaborate on that and discuss how this can address the current regulatory fragmentation in the EU on this matter.

**Authors' reply: We agree that this would be very interesting, but as this topic is very extensive, it would require further explanation that would need to be covered in another article.**

# Peer review: Reviewer response for version 1

**DOI:** 10.21956/openreseurope.14763.r27099

**Published:** 2021-07-19

**Authors:** Mark Taylor

**Affiliations:** 1 Melbourne Law School, Melbourne University, Melbourne, Australia

This is an interesting discussion that helps to illustrate the limitations of the GDPR when it comes to establishing regulatory harmonisation between Member States in relation to the processing of personal data for the purposes of health research. It provides interesting summaries of the regulatory position in four countries that are part of the EU/EEA (namely, Portugal, Finland, Norway and the Netherlands) and usefully signposts relevant primary material. 

 With regards to the scope of the scoping report, as the study was designed to investigate the possibility of linking cohort data with routine health and education data (comparing the four countries identified) (stated second column, page 4) it is not made clear why personal data derived from the analysis of biological samples is excluded from scope. It is stated that such personal data are excluded as a data category “as it deviates from the main objective of the study” (first column, page 5) but it is not made clear why.  It is not stated why personal data derived from analysis of biological samples are to be considered a separate and excluded category as a function of how they were derived.

 There are occasions when the legal analysis is not entirely clear or accurate. As a non-exhaustive list of illustrative examples,

1. The definition provided of personal information (toward the bottom of first column, page 4) does not correspond with the definition of personal data provided in the GDPR (Article 4(1)). There is a material difference with regards to the need for an individual to be identifiable *from *information classified as personal information (with GDPR defining personal data as data *relating to *an identified or identifiable natural person as personal information. To be personal data under the GDPR the data itself need not necessarily contribute toward identification).
2. The legal analysis occasionally moves back and forth between legal instruments produced by the European Union (such as the GDPR) and those produced by the Council of Europe (CoE) (such as the Recommendation No.R(97)18 of the Committee of Ministers to Members States concerning the protection of personal data collected and processed for statistical purposes) (see bottom of first para, page 11, fn 16-19) without acknowledging the distinction between EU and CoE and the nature of the interplay between these different sources of law.
3. Under the sub-heading ‘Legal basis for registries) it is stated (apparently in reference to GDPR) that “In order to access data, either an explicit informed consent provided in an oral or written format is required, or the anonymisation of personal data of the data subject” (column 2, page 11). The citation provided in support of this claim is “Article 9/1/a) [sic] GDPR; Recital 32 GDPR; Article 4/3 of 12/2005” (fn 23). There are a number of difficulties with this. The legal basis for processing is established in part by Article 6 GDPR, which is not referenced but provides more alternatives than consent.  Article 9 GDPR establishes a number of derogations to the general prohibition on the processing of personal data and the alternatives listed in Article 9 as permitting the processing of special categories of personal data also extend beyond consent. The Article 4/3 of 12/2005 cited is not fully referenced and so it is not clear what law is being referred to here. Table 3 “Main Involved Laws” provides only the reference Article 4/3 of 12/2005 Act, January 15, 2005. A more comprehensive analysis of GDPR is provided elsewhere but this illustrates one of the difficulties with the paper’s rather staccato structure.

There are also a number of typos (e.g. “sued” instead of “used”, toward top of second column page 4) and some odd syntax that can obscure meaning (e.g. in reference to Portugese law it is stated that the legal capacity of a person below the age of 18 “must be supressed through guardianship by holders of parental responsibility” (second column, page 13). I don’t know what that means, but it does not sound good!

 Despite some qualifications and reservations with regards to the legal analysis, the paper represents a useful contribution as a stimulation to debate, source of reference to national law, and comparison of the legal situation across four countries, helping to illustrate and confirm the limitations of the GDPR with regards to achieving harmonisation in this context.

Are the rationale for, and objectives of, the Systematic Review clearly stated?

Yes

Is the statistical analysis and its interpretation appropriate?

Not applicable

Are sufficient details of the methods and analysis provided to allow replication by others?

Partly

Are the conclusions drawn adequately supported by the results presented in the review?

Partly

Reviewer Expertise:

Health Law and Regulation

I confirm that I have read this submission and believe that I have an appropriate level of expertise to confirm that it is of an acceptable scientific standard, however I have significant reservations, as outlined above.

# Response

**Published:** 2021-09-17

**Authors:** Julia Nadine Doetsch

**Affiliations:** EPIUnit- Instituto de Saúde Pública da Universidade do Porto (ISPUP), Portugal

**The authors' responses to the reviewer report 1 were highlighted in bold.**

 This is an interesting discussion that helps to illustrate the limitations of the GDPR when it comes to establishing regulatory harmonisation between Member States in relation to the processing of personal data for the purposes of health research. It provides interesting summaries of the regulatory position in four countries that are part of the EU/EEA (namely, Portugal, Finland, Norway and the Netherlands) and usefully signposts relevant primary material. 

 With regards to the scope of the scoping report, as the study was designed to investigate the possibility of linking cohort data with routine health and education data (comparing the four countries identified) (stated second column, page 4) it is not made clear why personal data derived from the analysis of biological samples is excluded from scope. It is stated that such personal data are excluded as a data category “as it deviates from the main objective of the study” (first column, page 5) but it is not made clear why.  It is not stated why personal data derived from analysis of biological samples are to be considered a separate and excluded category as a function of how they were derived.

**Authors' reply: We would like to thank the reviewer Prof. Mark Taylor for their constructive feedback and thorough revision. With regards to your point raised on including biological samples, we had decided it would be too extensive. Therefore, this data category was excluded. We added a short explanation to the text: “and would involve an additional perspective that would lengthen the paper too extensively.”**

 There are occasions when the legal analysis is not entirely clear or accurate. As a non-exhaustive list of illustrative examples,

 1. The definition provided of personal information (toward the bottom of first column, page 4) does not correspond with the definition of personal data provided in the GDPR (Article 4(1)). There is a material difference with regards to the need for an individual to be identifiable *from *information classified as personal information (with GDPR defining personal data as data *relating to *an identified or identifiable natural person as personal information. To be personal data under the GDPR the data itself need not necessarily contribute toward identification).

**Authors' reply: We adapted the sentence to the exact quotation of Article 4(1) from the GDPR: “an information related to an identified or identifiable natural person [data subject].”**

 2. The legal analysis occasionally moves back and forth between legal instruments produced by the European Union (such as the GDPR) and those produced by the Council of Europe (CoE) (such as the Recommendation No.R(97)18 of the Committee of Ministers to Members States concerning the protection of personal data collected and processed for statistical purposes) (see bottom of first para, page 11, fn 16-19) without acknowledging the distinction between EU and CoE and the nature of the interplay between these different sources of law.

**Authors' reply: Thank you for this comment. We included the distinction between EU and CoE and the nature of the interplay between these sources of law. We added: “The Council of Europe (CoE), an international organisation encompassing 47 countries of Europe, was established to encourage democracy and guard human rights and the rule of law in Europe. The CoE Convention for the protection of individuals with regard to the processing of personal data (“Convention 108” of 28 January 1981) and additional protocols is a treaty that is open to any country (it currently counts 54 States). It provides for a legally binding commitment of countries with global dimension and it has inspired for decades a number of legislative processes throughout the world, such as for instance the EU data protection framework (namely the EU Data Protection Directive and the GDPR). The GDPR is an EU legal instrument that is a directly applicable and legally binding to the any EU/EEA Member States. The European Union presently has 27 members that have delegated some of their sovereignty so that decisions on certain issues of shared interest can be made democratically at European level.”**

 3. Under the sub-heading ‘Legal basis for registries) it is stated (apparently in reference to GDPR) that “In order to access data, either an explicit informed consent provided in an oral or written format is required, or the anonymisation of personal data of the data subject” (column 2, page 11). The citation provided in support of this claim is “Article 9/1/a) [sic] GDPR; Recital 32 GDPR; Article 4/3 of 12/2005” (fn 23). There are a number of difficulties with this. The legal basis for processing is established in part by Article 6 GDPR, which is not referenced but provides more alternatives than consent.  Article 9 GDPR establishes a number of derogations to the general prohibition on the processing of personal data and the alternatives listed in Article 9 as permitting the processing of special categories of personal data also extend beyond consent. The Article 4/3 of 12/2005 cited is not fully referenced and so it is not clear what law is being referred to here. Table 3 “Main Involved Laws” provides only the reference Article 4/3 of 12/2005 Act, January 15, 2005. A more comprehensive analysis of GDPR is provided elsewhere but this illustrates one of the difficulties with the paper’s rather staccato structure.

**Authors' reply: Thank you. We adapted the text to: “In order to access data, a legal basis is needed according to the GDPR (Articles 6 and 9). Member States may introduce further conditions with regards to the processing of health data.” The article 4/3 of 12/2005 was removed as by mistake national Portuguese law was included. We instead included article 9 of the GDPR. Table 3, Article 4/3 of 12/2005 Act, January 15, 2005. This is the Portuguese law that is also referred to in the comparative section. It was moved to the Portuguese section. With regards to the paper’s structure, we chose this structure to ease comparison between countries. There are also a number of typos (e.g. “sued” instead of “used”, toward top of second column page 4) and some odd syntax that can obscure meaning (e.g. in reference to Portuguese law it is stated that the legal capacity of a person below the age of 18 “must be supressed through guardianship by holders of parental responsibility” (second column, page 13). I don’t know what that means, but it does not sound good! Authors' reply: Thank you, we corrected the typos. The sentence “must be suppressed through guardianship by holders of parental responsibility” was adapted to “It requires the authorization or intervention through guardianship of the holders of parental responsibility” to be more neutral and avoid misunderstandings.**

 Despite some qualifications and reservations with regards to the legal analysis, the paper represents a useful contribution as a stimulation to debate, source of reference to national law, and comparison of the legal situation across four countries, helping to illustrate and confirm the limitations of the GDPR with regards to achieving harmonisation in this context.

**Authors' reply: We thank the reviewer for his comprehensive feedback and constructive comments. We have adapted the paper accordingly.**
